# Histone H1.0 couples cellular mechanical behaviors to chromatin structure

**DOI:** 10.1038/s44161-024-00460-w

**Published:** 2024-04-10

**Authors:** Shuaishuai Hu, Douglas J. Chapski, Natalie D. Gehred, Todd H. Kimball, Tatiana Gromova, Angelina Flores, Amy C. Rowat, Junjie Chen, René R. Sevag Packard, Emily Olszewski, Jennifer Davis, Christoph D. Rau, Timothy A. McKinsey, Manuel Rosa-Garrido, Thomas M. Vondriska

**Affiliations:** 1 Department of Anesthesiology & Perioperative Medicine, David Geffen School of Medicine at University of California, Los Angeles, Los Angeles, CA USA; 2 Department of Integrative Biology and Physiology, University of California, Los Angeles, Los Angeles, CA USA; 3 Department of Medicine, Division of Cardiology, David Geffen School of Medicine at University of California, Los Angeles, Los Angeles, CA USA; 4 Department of Physiology, David Geffen School of Medicine at University of California, Los Angeles, Los Angeles, CA USA; 5 Department of Bioengineering, University of Washington, Seattle, WA USA; 6 Department of Genetics and McAllister Heart Institute, University of North Carolina, Chapel Hill, NC USA; 7 Department of Medicine, Division of Cardiology and Consortium for Fibrosis Research & Translation, University of Colorado Anschutz Medical Campus, Aurora, CO USA; 8 Department of Biomedical Engineering, School of Medicine and School of Engineering, University of Alabama at Birmingham, Birmingham, AL USA

**Keywords:** Cell biology, Physiology, Cardiovascular diseases, Histone post-translational modifications

## Abstract

Tuning of genome structure and function is accomplished by chromatin-binding proteins, which determine the transcriptome and phenotype of the cell. Here we investigate how communication between extracellular stress and chromatin structure may regulate cellular mechanical behaviors. We demonstrate that histone H1.0, which compacts nucleosomes into higher-order chromatin fibers, controls genome organization and cellular stress response. We show that histone H1.0 has privileged expression in fibroblasts across tissue types and that its expression is necessary and sufficient to induce myofibroblast activation. Depletion of histone H1.0 prevents cytokine-induced fibroblast contraction, proliferation and migration via inhibition of a transcriptome comprising extracellular matrix, cytoskeletal and contractile genes, through a process that involves locus-specific H3K27 acetylation. Transient depletion of histone H1.0 in vivo prevents fibrosis in cardiac muscle. These findings identify an unexpected role of linker histones to orchestrate cellular mechanical behaviors, directly coupling force generation, nuclear organization and gene transcription.

## Abstract (web summary)

Hu et al. describe how histone H1.0 regulates myofibroblast activation, linking force generation to nuclear organization and gene transcription.

## Main

The varied physiological demands of different organ systems necessitate coping with a wide range of mechanical forces and extracellular signals. Fibroblasts are a specialized cell type present across most mammalian tissues that are responsible for synthesis of connective tissue. In adulthood, the actions of fibroblasts are essential to maintain tissue integrity and to respond to injury or cell death in various organs through a process that involves adoption of a myofibroblast phenotype. Activated myofibroblasts develop actin stress fibers, become contractile and synthesize extracellular matrix (ECM) as part of a response that stiffens the tissue and heals wounds^1^. The plasma membrane is connected to the ECM by a network of proteins that tethers the cell within the organ, thereby facilitating communication between cells and relaying extracellular physical cues to the intracellular organelles. In situations of stress, extracellular cues in the form of physical forces, cytokines and hormones induce changes in transcription that alter the mechanical properties of the cell.

The nucleus itself has been shown to directly influence distensibility of the cell and respond to mechanical signals^2^. The nucleoskeleton is coupled to the cellular cytoskeleton, enabling force transduction and physical regulation of cellular compliance. Changes in nuclear deformability and histone post-translational modification have been shown to adaptively respond to mechanical stress, protecting the genome against aberrant gene expression^3^. Changes in extracellular tension can directly impact nuclear flexibility and chromatin compaction in fibroblasts^4^, and the general compaction state of chromatin can influence the mechanical stability and activation state of the cell^5^. The functional unit of chromatin is an octamer of two copies each of histone H2A, H2B, H3 and H4 wrapped with approximately 145–147 base pairs of DNA, together comprising a nucleosome^6^. Chromatin is then packaged into higher-order structures, ranging from fibers comprising dozens of nucleosomes and a few kilobases of DNA to topologically associated domains (thousands of nucleosomes and megabases of DNA) and nuclear territories (whole chromosomes)^7^. The complex packaging rules that govern how the same genome is stored and retrieved differently across cells are known to involve the actions of histone-modifying proteins, which post-translationally modify core histones, thereby priming the targeted regions of chromatin for tasks such as DNA repair, replication, transcription and gene silencing^8,9^. However, chromatin has unexpected functions beyond genome packaging—for example, acting as a lens in rod cells of the eyes of nocturnal animals^10^ and functioning as a copper reductase^11^. We reasoned chromatin may control nuclear—and, thereby, cellular—compliance, and we set out to identify a molecular regulator of such activity. We focused on the linker histone H1 family of proteins, given their role in promoting chromatin folding^12^.

The mouse linker histone H1 family comprises five (H1.1–1.5) main isoforms, plus the oocyte-specific H1oo, the testis-specific H1t and the replacement variant H1.0. Linker histones bind the nucleosome, facilitating chromatin compaction and the formation of higher-order structures comprising multiple nucleosomes and associated DNA^13^. Loss-of-function studies have shown that individual histone H1 isoforms are dispensable for normal mouse development^14,15^, yet triple knockouts (deleting H1.3, H1.4 and H1.5) showed extensive developmental abnormalities^16^, associated with an altered linker/core histone ratio, which was maintained when only one isoform was deleted via compensatory upregulation of other isoforms. These findings highlight the central role of histone stoichiometry in controlling normal development and tissue homeostasis^12^. In cancer cells, altering histone H1 levels substantially reorganized global chromatin structure, decompacting topologically associating domains^17^ and shifting the genome to a more relaxed state^18^.

Fibroblast activation leads to expression of cytoskeletal and ECM genes through a process that requires the activity of histone-modifying enzymes, including histone deacetylases (HDACs), and chromatin readers, including BRD4 (ref. ^19^). Furthermore, the ability of chromatin-remodeling enzymes to modulate gene expression can be influenced by the local topology of chromatin^20^, which may be regulated by the abundance of linker histone H1. A fundamental unanswered question is how the cell processes stress signals at the nucleus to remodel chromatin for precise gene expression, integrating the nucleosome-targeted actions of chromatin remodeling machinery and the genome-sculpting behavior of chromatin structural proteins, to elicit different mechanical responses.

We hypothesized that linker histone H1 may participate in this process of fibroblast stress response by changing the packaging of the genome. We found that levels of histone H1.0 underpin a genome-wide change in chromatin organization to facilitate transcriptional changes in cytoskeletal and ECM genes. We show that histone H1.0 is required for fibroblast activation in response to cytokine stimulation and that overexpression of histone H1.0 is sufficient to activate fibroblasts in the absence of stimulation. Histone H1.0 acts locally to promote formation of more compact chromatin fibers and globally to condense the genome, in turn regulating cellular deformability. Histone H1.0 is required for cytokine-induced reprogramming of the activating chromatin modification histone H3 lysine 27 acetylation (H3K27Ac) and acts via modulation of HDACs and BRD4. Finally, we demonstrate that these chromatin regulatory actions of histone H1.0 affect a wide range of mechanical behaviors in the cell, including contractile force generation, cytoskeletal regulation, motility and ECM deposition.

## Results

### Histone H1.0 is enriched in fibroblasts and stress responsive

**Fig. 1:**
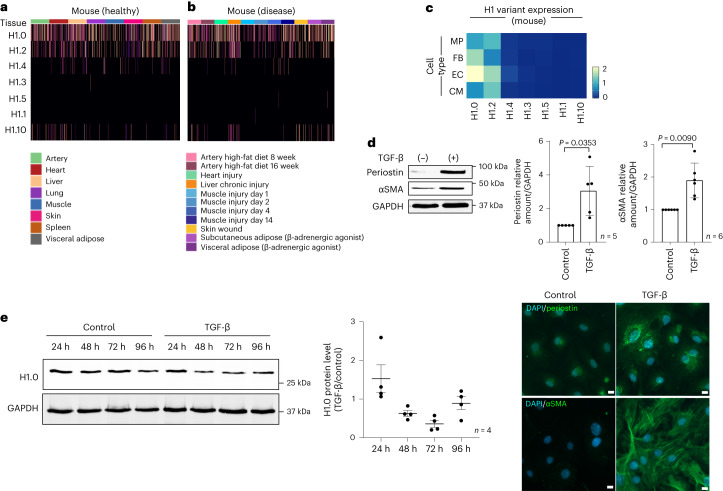
Histone H1.0 is the principal histone H1 isoform in mouse fibroblasts. **a**,**b**, Heatmaps showing expression of H1 isoforms in fibroblasts from healthy murine tissue (**a**) and murine disease models (**b**). Data from https://www.fibroxplorer.com/ (Buechler et al.^21^). **c**, Heatmap of scRNA-seq data (Ren et al.^24^) showing average expression of each H1 isoform in murine cardiac cell types. MP, macrophage; FB, fibroblast; EC, endothelial cell; CM, cardiomyocyte. **d**, Top left: αSMA and periostin expression after TGF-β treatment as measured by western blot. Top right: quantification of western blot (mean ± s.d.; Welch’s unpaired *t*-test). Bottom: periostin and αSMA immunostaining in fibroblasts after TGF-β (10 ng ml^−1^, 48 h) or vehicle control (nuclei stained with DAPI; scale bars, 10 µm; representative of *n* = 4 biological replicates from separate isolations; mean ± s.d.; Welch’s unpaired *t*-test). **e**, Left: western blot of histone H1.0 protein abundance after fibroblast activation (24 h, 48 h, 72 h and 96 h, 10 ng ml^−1^ TGF-β). Right: immunoblot quantification (TGF-β signal as fold of control; mean ± s.e.m.; no significant differences; one replicate in **d** and **e** is a single isolation followed by treatment as indicated and western blotting). Source data

**Extended Data Fig. 1:**
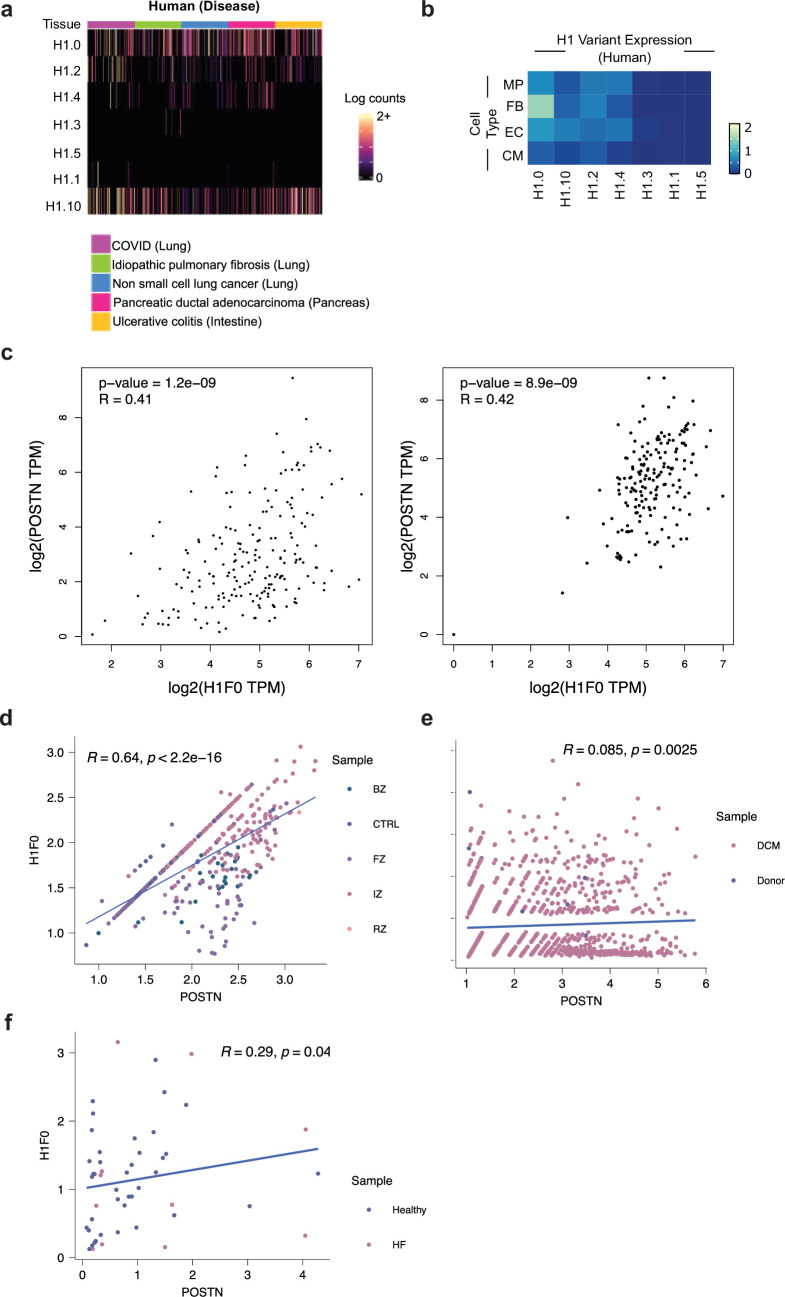
Relationship between histone H1.0 and periostin expression in human datasets. **a**) Heatmaps showing expression of H1 isoforms in fibroblasts from human disease models. Data from fibroXplorer.com (Buechler et al. ^21^). **b**) Heatmaps showing average expression of each H1 isoform in macrophages (MP), fibroblasts (FB), endothelial cells (EC) and cardiomyocytes (CM) from single-cell RNA-seq analysis of healthy human hearts (Wang et al. ^23^). **c**) A significant positive correlation was observed between H1F0 (histone H1.0) and POSTN (periostin) mRNA expression in the GTEx database, as calculated using the GEPIA web server (Tang et al., Nucleic Acids Res. 2017;45:W98-W102) in both the human left ventricle (left) and left atrial appendage (right). Spearman correlation coefficients and p-values are shown. **d**) A positive spearman correlation was also observed between H1F0 and POSTN mRNA expression in the fibroblast subset of a single nucleus RNA-seq dataset from human myocardial infarcted tissue (Kuppe et al., Nature 2022;608:766–777). Normalized counts of H1F0 and POSTN are plotted from the 345 cardiac fibroblasts that express non-zero transcripts of both genes, colored by region of origin (BZ = Border Zone, CTRL = Control, FZ = Fibrotic Zone, IZ = Ischemic Zone, RZ = Remote Zone). **e**) Spearman correlation between H1F0 and POSTN mRNA expression as calculated from the fibroblast subset of a single cell RNA-seq dataset from dilated cardiomyopathy or donor human hearts (Koenig et al. Nat Cardiovasc Res. 2022;1:263–280). **f**) Spearman correlation between POSTN and H1F0 mRNA expression calculated from the fibroblast subset of a single nucleus RNA-seq dataset from human heart failure patients or healthy controls (Wang et al. Nat Cell Biol. 2020;22:108–119). Linear regression line is shown in blue, and p-value was calculated using cor.test() in R for panels (**c-f**).

**Extended Data Fig. 2:**
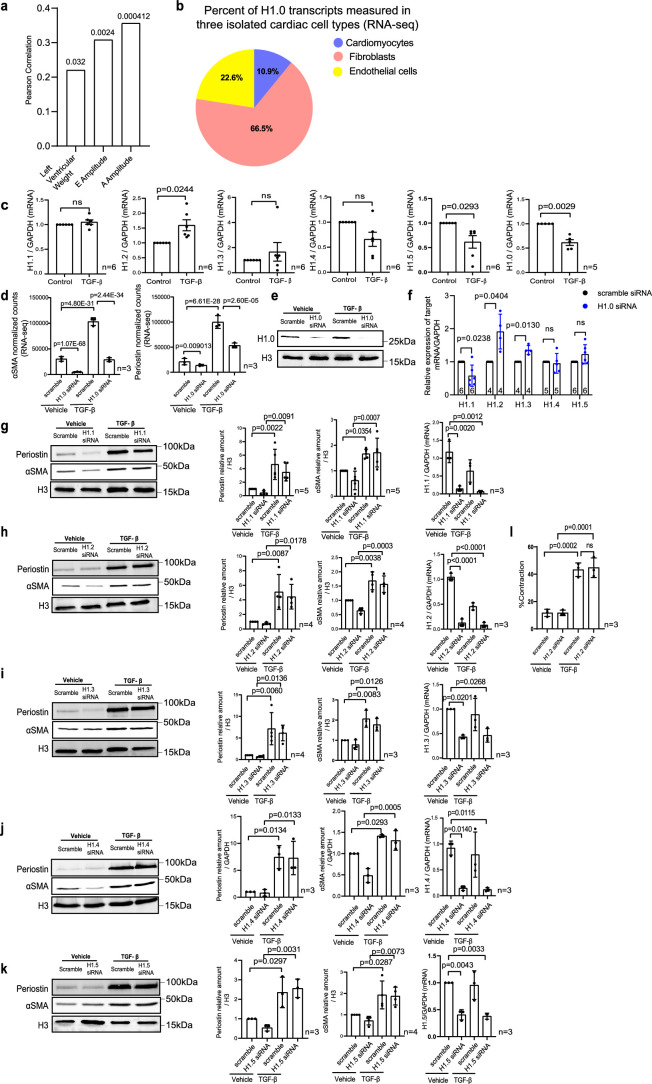
Histone H1.0 is enriched in fibroblasts, associated with cardiac dysfunction and the only isoform whose depletion influences fibroblast activation. **a**) Bar chart showing significant Pearson correlation (indicated on y-axis) between cardiac H1.0 transcription in the Hybrid Mouse Diversity Panel (Rau et al. Circ CV Genetics, 2015) and left ventricular weight, E amplitude, and A amplitude. For all three phenotypes, p-values (top of bars) were calculated using cor.test() in R. Transcription and phenotype data were measured in hearts from control mice. **b**) RNA-seq data from adult mouse hearts, examining transcription in isolated myocytes, fibroblasts, and endothelial cells. **c**) RT-qPCR on histone H1 isoforms performed in isolated murine cardiac fibroblasts activated by TGF-β (48 h, 10 ng/mL; mean ± SEM, Welch’s unpaired t-test). **d**) RNA-seq shows that siRNA knockdown of histone H1.0 significantly inhibits TGF-β induced αSMA (*left*) and periostin (*right*) transcription (mean ± SD, Benjamin’s-Hochberg adjusted Wald test p-value from the DESeq2 package is shown). **e**) Western blotting shows histone H1.0 KD does not affect histone H3 protein level (representative of n = 3 biological replicates). **f**) Graph showing RT-qPCR of all remaining H1 isoforms after H1.0 siRNA depletion (mean ± SD, Welch’s unpaired t-test for each H1 isoform). **g-k**) Western blots and quantitation of periostin, αSMA and the indicated histone H1 isoforms following TGF-β in the presence or absence of knockdown of the various histone H1 family members (mean ± SD, one-way ANOVA with a post-hoc Tukey test). **l**) Quantification of collagen gel contraction (one replicate is an individual isolation and single well area calculation; same as in Fig. 2d) following TGF-β treatment in the presence or absence of histone H1.2 knockdown (mean ± SD, one-way ANOVA with a post-hoc Tukey test). Source data

To investigate the role of linker histone isoforms in response to cellular stress, we examined single-cell RNA sequencing (scRNA-seq) data^21^ to reveal the natural variation in these isoforms among cells. Because fibroblasts are ubiquitous cells present in nearly all organs of the body, we examined a database of murine fibroblasts from various organs and found that, regardless of tissue of origin, linker histone H1.0 is more highly expressed than other linker histone H1 variants (Fig. 1a). Fibroblasts play an important role in sensing extracellular tension and responding to organ-level stress: we therefore further examined linker histone variant transcripts in fibroblasts from various injured murine tissues. In the mouse tissues surveyed, H1.0 is the predominant linker histone variant (Fig. 1b). Whether the same histone isoforms are operative in humans is unclear: histone H1.0 and H1.10 are expressed in fibroblasts from diseased human tissues (Extended Data Fig. 1a,b), and analysis of three separate scRNA-seq datasets from human hearts revealed a positive correlation between histone H1.0 and periostin, a canonical marker of fibroblast activation, as did data from the Genotype-Tissue Expression (GTEx) project (Extended Data Fig. 1c–f). Global transcriptome analyses in a genetically diverse population of mice administered the adrenergic agonist isoproterenol (ISO)^22^, which stiffens the muscle through increased fibrosis, showed a strong association of histone H1.0 levels with metrics of heart muscle pathology and dysfunction, including left ventricular (LV) mass and the echocardiography parameters E and A amplitude, measurements of the heart’s ability to relax during diastole (Extended Data Fig. 2a). Bulk RNA sequencing (RNA-seq) analyses of mouse hearts showed that, despite cardiomyocytes contributing the vast majority of the heart mass, fibroblasts accounted for the greatest level of histone H1.0 transcript expression (Extended Data Fig. 2b), a finding also supported by analysis of scRNA-seq data of murine cardiac cells (Fig. 1c). Histone H1.0 is known to be the principal, if not the only, histone H1 isoform poly-adenylated in mammals. Figure 1a,b depicts data obtained from the poly(A) capture of mRNAs^21^, revealing histone H1.0 and H1.2 to be the most abundant at the transcript level. However, Extended Data Fig. 1b,c shows data that are not based on poly(A) capture of mRNA^23,24^, wherein ribosomal RNA depletion was employed, leading to the same observation. Thus, we reason that the greater abundance of histone H1.0 at the transcript level is not due to a bias of poly(A) selection. Our previous work also indicates that this differential abundance is reflected at the protein level^25^.

To test this role of histone H1.0 in fibroblast activation, we adopted a primary adult mouse fibroblast model system treated with transforming growth factor beta (TGF-β), a cytokine involved in stress response throughout the body, including in the heart^26^. TGF-β treatment induced fibroblast activation as measured by periostin and alpha smooth muscle actin (αSMA) protein expression and demonstrated by actin stress fiber formation (Fig. 1d), concomitant with dynamic changes in histone H1.0 protein levels over time (Fig. 1e). Histone H1.2, the second-most abundant isoform in fibroblasts, was increased in abundance at 48 h after TGF-β, whereas H1.5 was decreased (Extended Data Fig. 2c). All other isoforms of histone H1 were unaffected by TGF-β treatment (Extended Data Fig. 2c).

### Histone H1.0 is necessary and sufficient for fibroblast activation

**Fig. 2:**
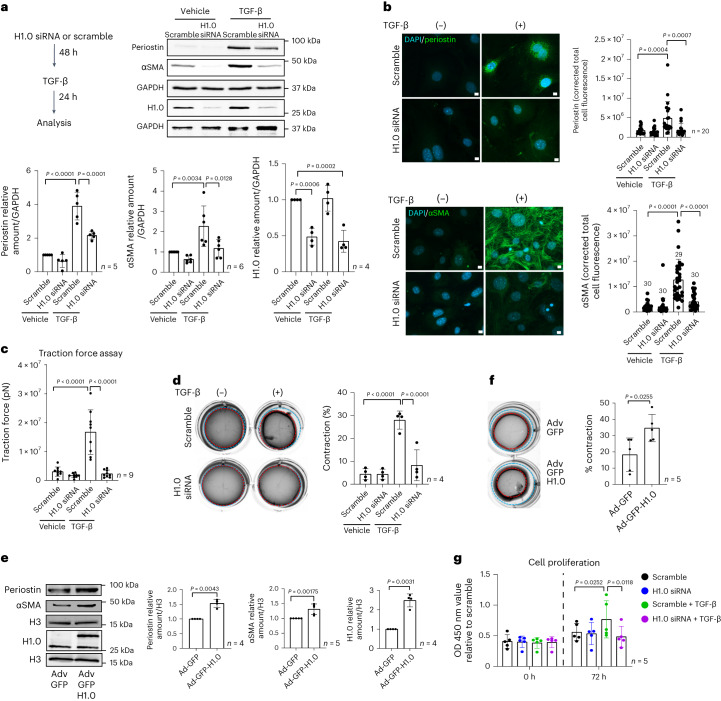
Histone H1.0 is necessary for stress-induced activation of fibroblast mechanical behaviors. **a**, Treatment schematic (top left). αSMA and periostin protein levels after TGF-β treatment and effect of histone H1.0 KD (top right), along with quantification (bottom; one-way ANOVA post hoc Tukey test; mean ± s.d.; one replicate is a single isolation followed by treatment as indicated and western blotting). **b**, Immunofluorescence and quantification of periostin (top) and αSMA (bottom) protein localization in situ (DAPI stains cell nuclei; scale bars, 10 µm; mean ± s.d.). **c**, Traction force assay measuring force production at the single-cell level (one replicate is an individual isolation and contraction force measurement). **d**, Gel contraction assay (one replicate is an individual isolation and single well area calculation; one-way ANOVA post hoc Tukey test; mean ± s.d.). **e**, Western blot demonstrating effect of histone H1.0 overexpression on periostin and αSMA protein levels (left) and quantification (right three panels; Welch’s unpaired *t*-test; mean ± s.d.; one replicate is a single isolation followed by treatment as indicated and western blotting). **f**, Histone H1.0 overexpression and effect on gel contraction (left) and quantification (right; Welch’s unpaired *t*-test; mean ± s.d.; one replicate is an individual isolation and single well area calculation). **g**, Absorbance measurements at 450 nm wavelength measure cell proliferation (mean ± s.d. with one-way ANOVA with a post hoc Tukey test; one replicate is a single isolation followed by treatment as indicated and calculation of cell number). KD, knockdown; OD, optical density. Source data

Previous studies demonstrated compensatory upregulation of other isoforms after germline deletion of individual linker histones^14,15^. We therefore used an siRNA-mediated knockdown approach targeting the six main isoforms expressed in somatic cells. Knockdown of histone H1.0 before administration of the cytokine was sufficient to prevent TGF-β-induced fibroblast activation as measured by periostin and αSMA transcript (Extended Data Fig. 2d) and protein abundance (Fig. 2a). Depletion of histone H1.0 also prevented actin stress fiber formation (Fig. 2b). Knockdown of histone H1.0 had modest effects on other H1 isoforms (Extended Data Fig. 2f), yet individual knockdown of the other five isoforms had no effect on fibroblast activation (Extended Data Fig. 2g–k; note: knockdown of histone H1.2, the next-most abundant H1 isoform, does not affect TGF-β-induced gel contraction; Extended Data Fig. 2l), illustrating that, even though genetic loss of linker histone H1 isoforms can be compensated developmentally^14,16^, these individual isoforms play distinct roles in the somatic cell.

**Extended Data Fig. 3:**
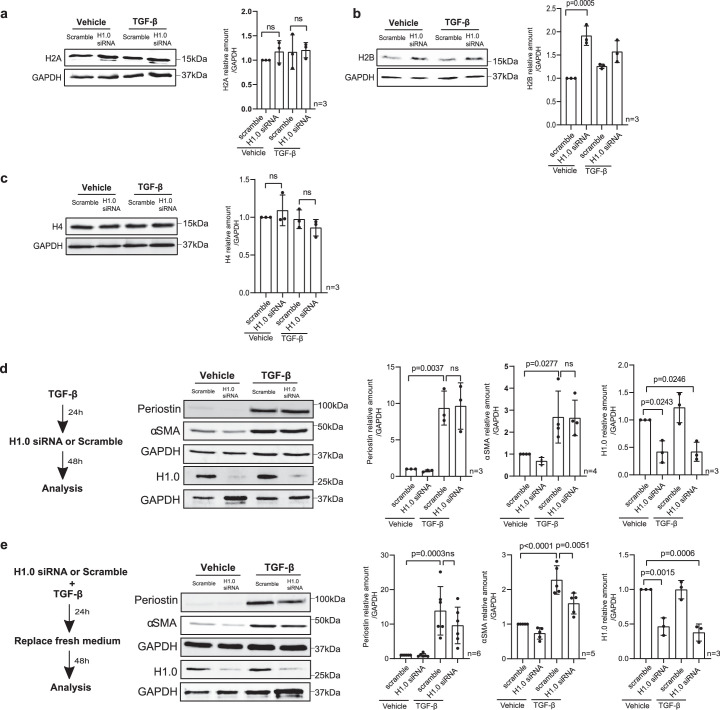
Effect of histone H1.0 depletion on TGF-β-dependent fibroblast activation. **a-c**) Western blot and quantitation of effect of TGF-β and histone H1.0 KD on histone H2A, H2B and H4. **d**) Knockdown of histone H1.0 after fibroblast activation with TGF-β fails to block αSMA and periostin induction (n = 3). **e**) Concomitant histone H1.0 KD and TGF-β treatment slightly attenuates αSMA induction. All data are mean ± SD, analyzed by one-way ANOVA with a post-hoc Tukey test; one replicate in **a-e** is a single isolation followed by treatment as indicated and western blotting. Source data

Depletion of histone H1.0 did not alter levels of core histones H3, H2A or H4, with only a modest change in the level of H2B (Extended Data Figs. 2e and 3a–c), thereby resulting in a decreased linker/core ratio. These observations are in contrast to germline knockouts of H1 isoforms, which result in compensatory alteration in other core histone levels and a maintenance of the linker-to-core nucleosome ratio—a key feature shown previously to regulate chromatin structure and nuclear architecture^12,27^. Previous studies observed fewer linker histones per nucleosome in the setting of cardiac hypertrophy^25^. These findings suggest that transient depletion of histone H1.0 alters the linker/core histone ratio, unmasking endogenous roles of linker histones that are compensated for in histone H1.0 germline knockouts. If the fibroblasts are already activated, histone H1.0 knockdown does not reverse the effects of TGF-β (Extended Data Fig. 3d), whereas simultaneous knockdown at the time of TGF-β treatment was sufficient to block αSMA but not periostin expression (Extended Data Fig. 3e). We hypothesize that proper histone stoichiometry is necessary for stress response in fibroblasts and that, once the transcriptional program is activated in response to agonist, the window for modulating chromatin architecture to prevent this stress response has closed.

**Extended Data Fig. 4:**
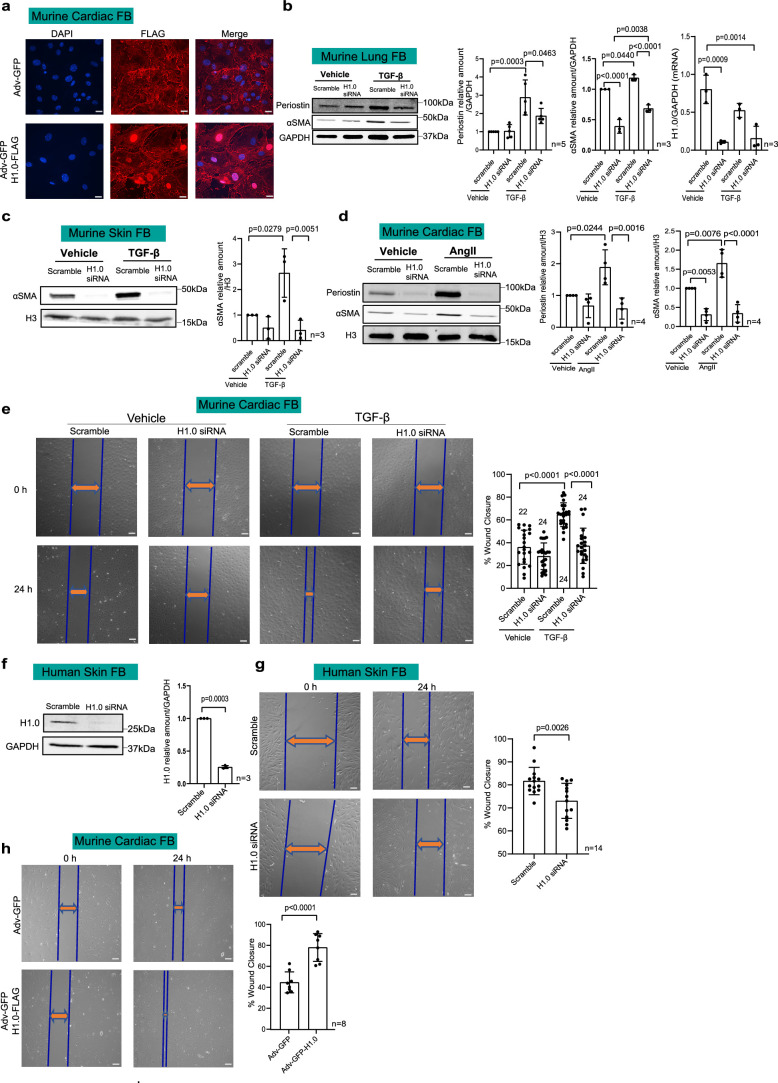
Depletion of histone H1.0 prevents distinct fibroblast mechanical behaviors in cells from different organs and in response to different stimuli. **a**) Murine cardiac fibroblasts transfected with Adv-GFP-H1.0-FLAG of Adv-GFP control (48 h) were immunolabeled with an anti-FLAG antibody to show nuclear localization of histone H1.0 (*red*). DAPI (*blue*) stains cell nuclei. Scale bar=10μm (n = 3 independent experiments). **b**) *Left*, Western blot in isolated murine lung fibroblasts transfected with histone H1.0 siRNA or scrambled negative control (48 h) and then treated with TGF-β shows upregulation of periostin and αSMA is histone H1.0 dependent in lung. *Center left*, Immunoblot quantification. *Center right*, Efficiency of histone H1.0 KD is demonstrated by RT-qPCR (mean ± SD, one-way ANOVA with a post-hoc Tukey test). **c**) *Left*, Western blot in isolated murine skin fibroblasts transfected with histone H1.0 siRNA or scrambled negative control (48 h) and then treated with TGF-β shows up-regulation of αSMA is histone H1.0 dependent in skin. *Right*, Immunoblot quantification (mean ± SD; one-way ANOVA with a post-hoc Tukey test). **d**) *Left*, Western blot in isolated murine cardiac fibroblasts transfected with histone H1.0 siRNA or scrambled negative control (48 h) and then treated with Angiotensin II (48 h, 1 µM) shows that induction of periostin and αSMA upregulation by Angiotensin II is histone H1.0 dependent. *Middle and right*, Immunoblot quantification (mean ± SD, one-way ANOVA with a post-hoc Tukey test; one replicate in **b-d** is a single isolation followed by treatment as indicated and western blotting). **e**) *Left*, A wound scratch assay was conducted using murine cardiac fibroblasts, demonstrating the necessary role of histone H1.0 in TGF-β-induced cell migration and proliferation. A scratch wound was made across the cell layer as indicated by the guidelines. Images were captured along the scratch wound guidelines at 0 h and 24 h. *Right*, Percentage of wound closure ([0 h wound area – 24 h wound area] / 0 h wound area) was measured using ImageJ (mean ± SD, one-way ANOVA with a post-hoc Tukey test). **f**) *Left*, Histone H1.0 knockdown confirmed by western blot in human skin fibroblasts. *Right*, quantification (mean±SD, Welch’s unpaired t-test). **g**) *Left*, Histone H1.0 knockdown impairs human skin fibroblast migration, *Right*, Quantification. **h**) *Left*, Wound scratch assay using murine cardiac fibroblasts transfected with Adv-GFP-H1.0-FLAG or Adv-GFP control (*right*, quantification: 48 h; mean ± SD, Welch’s unpaired t-test). Mean ± SD, Welch’s unpaired t-test; one replicate in **e-h** is a single isolation followed by treatment as indicated and calculation of cell migration). Source data

To test whether changes in marker gene expression were indicative of phenotypic changes in activated fibroblasts, we examined the effect of histone H1.0 depletion on distinct mechanical behaviors in primary cells. We employed a traction force assay, in which cells were seeded onto fluorescently labeled BSA beads, and the deformation of the beads was used to measure the force generated by individual fibroblasts^28^. Treatment with TGF-β induced robust traction force generation at the individual cell level, and this response was completely abrogated by depletion of histone H1.0 (Fig. 2c; note: in traction force experiments, which measure single cells, all groups were co-transfected with an siRNA conjugated to Cy3 fluorescent tag along with either the scrambled siRNA or the siRNA against histone H1.0, and only cells expressing this tag were selected for measurement). When the contractile behavior of the whole population of cells on the culture dish was examined with a gel contraction assay, histone H1.0 was again found to be necessary for the TGF-β-induced contractile phenotype (Fig. 2d). Furthermore, overexpression of histone H1.0 demonstrated that increasing the levels of this protein in the nucleus (Extended Data Fig. 4a) was sufficient to induce fibroblast activation in the absence of cytokine stimulation as measured by periostin and αSMA expression (Fig. 2e) and gel contraction assay (Fig. 2f).

To examine whether the relationship between histone H1.0 and fibroblast mechanical behaviors was more universal, we used a different activating stimulus to examine distinct mechanical properties of cells from various tissues and species. Histone H1.0 was necessary for activation of fibroblasts from mouse lung (Extended Data Fig. 4b), mouse skin (Extended Data Fig. 4c) or human skin (Extended Data Fig. 4f,g). Activation of cardiac fibroblasts by angiotensin II was also dependent on histone H1.0 (Extended Data Fig. 4d). Depletion of histone H1.0 does not impair cell viability but was sufficient to prevent cardiac fibroblast proliferation in response to TGF-β (Fig. 2g), as measured by CCK-8 viability assay, as well as in the setting of a cell migration assay in which confluent cells are mechanically disrupted and allowed to close a pseudo wound (Extended Data Fig. 4e,g). Overexpression of histone H1.0 promoted active wound closure in the absence of TGF-β stimulation (Extended Data Fig. 4h), demonstrating that histone H1.0 is sufficient to induce this proliferative response.

### Histone H1.0 coordinates chromatin remodeling machinery

**Fig. 3:**
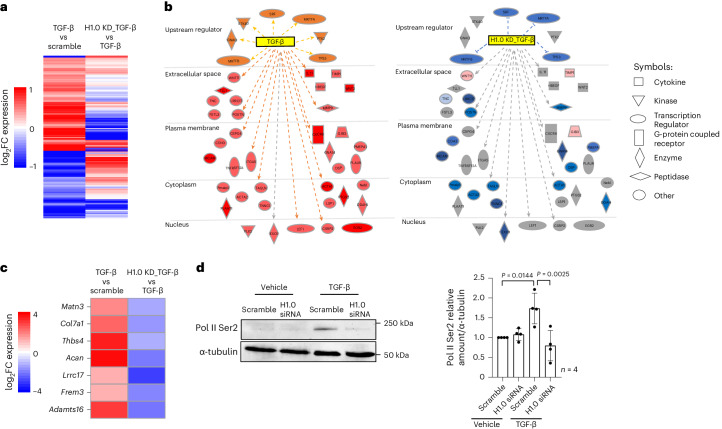
Histone H1.0 is necessary for transcriptional activation in response to TGF-β. **a**, Heatmap of gene expression changes TGF-β versus scramble (left) and TGF-β + H1.0 KD versus TGF-β (right; a subset of genes with *P* < 0.01 was selected; Benjamini–Hochberg-adjusted Wald test *P* value from the DESeq2 package). **b**, IPA identified genes significantly altered by TGF-β stimulation (left) and those whose expression is influenced by histone H1.0 KD before TGF-β (right). Upregulated genes are shown (left) (orange shading indicates activation; light/dark red indicates increased transcription after TGF-β treatment; and orange lines represent predicted and measured activation). Expression of these same genes when histone H1.0 is depleted prior to TGF-β versus TGF-β alone is shown on the right, showing widespread inhibition of transcriptional changes (light/dark blue indicates decreased expression; light red indicates increased expression; and gray indicates no change). Blue lines indicate predicted and measured inhibition. Gray lines indicate no prediction of direction. **c**, Heatmap showing effect of histone H1.0 KD on the expression pattern of ECM genes activated by TGF-β treatment. **d**, Western blot shows effects of histone H1.0 KD on TGF-β-induced changes in RNA Pol II Ser2 phosphorylation (mean ± s.d., analyzed by one-way ANOVA with a post hoc Tukey test; one replicate is a single isolation followed by treatment as indicated and western blotting). FC, fold change; KD, knockdown. Source data

**Extended Data Fig. 5:**
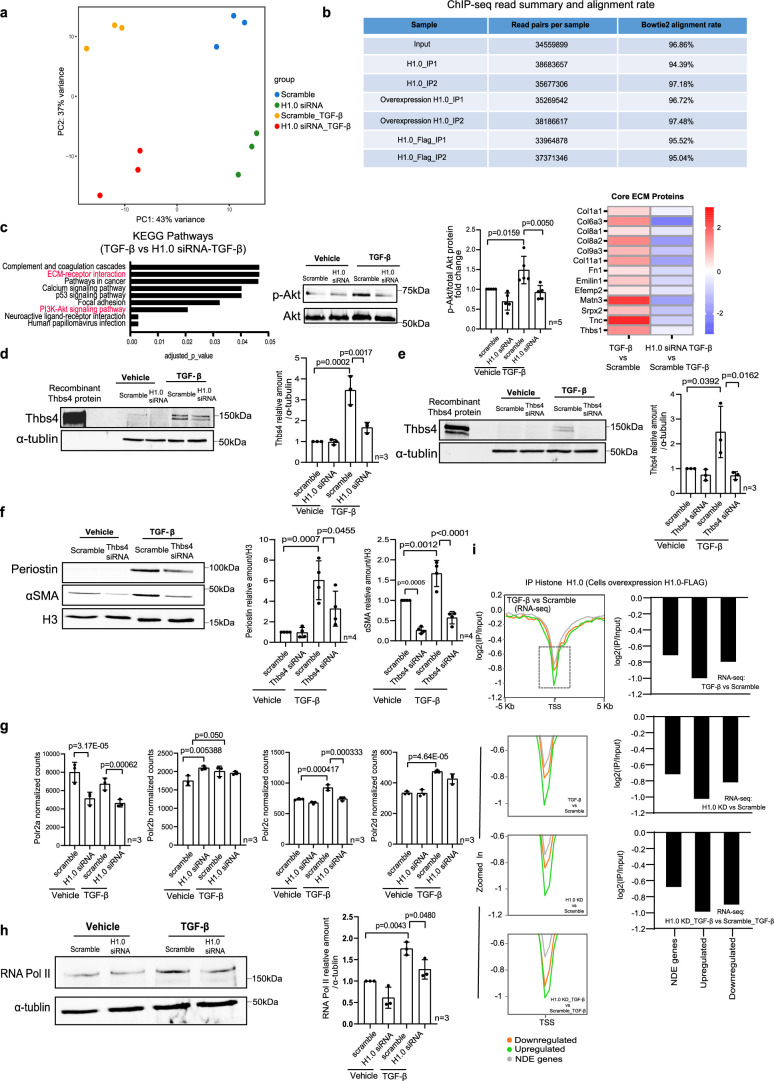
Effects of histone H1.0 depletion of transcriptional machinery and stress responsive genes. **a**) Principal component analysis of RNA-seq data (3 biological replicates per condition). PC1 captures effect of TGF-β, PC2 of histone H1.0 KD. **b**) Table summarizing ChIP-seq read pair counts and mapping rates for each replicate. **c**) *Left*, KEGG pathway analysis of TGF-β-upregulated genes that were histone H1.0 dependent. *Center left*, Western blot of p-Akt and total Akt in isolated murine cardiac fibroblasts following TGF-β treatment in the presence or absence of histone H1.0 KD. *Center right*, Immunoblot quantification (mean ± SD, one-way ANOVA with a post-hoc Tukey test). *Right*, Extended analysis of collagens and other ECM genes reveals dependency of their upregulation on histone H1.0. **d**) *Left*, Western blot showing that histone H1.0 KD abrogates TGF-β-induced upregulation of the extracellular matrix protein thrombospondin 4 (THBS4). *Right*, Immunoblot quantification (mean ± SD, one-way ANOVA with a post-hoc Tukey test). **e**) *Left*, Western blot confirming Thbs4 knockdown in primary cardiac fibroblasts. *Right*, Immunoblot quantification (mean n ≥ 3 ± SD, one-way ANOVA with a post-hoc Tukey test). **f**) *Left*, Western blot showing Thbs4 KD prevents induction of periostin and αSMA by TGF-β. *Right*, Immunoblot quantification (mean ± SD, one-way ANOVA with a post-hoc Tukey test; one replicate in **c-e** is a single isolation followed by treatment as indicated and western blotting). **g**) Transcript levels of four RNA Polymerase II subunits was analyzed by RNA-seq (mean ± SD, Benjamin’s-Hochberg adjusted Wald test p-value from the DESeq2 package is shown; one replicate is one RNA-seq experiment). **h**) *Left*, Western blot demonstrating effect of histone H1.0 KD on TGF-β induced RNA Pol II upregulation. *Right*, Immunoblot quantification (mean ± SD, one-way ANOVA with a post-hoc Tukey test; one replicate is a single isolation followed by treatment as indicated and western blotting). **i**) *Left panels*, Histone H1.0 ChIP-seq, performed using an antibody against endogenous histone H1.0 in isolated cardiac fibroblasts overexpressing histone H1.0-FLAG, reveals depletion of H1.0 occupancy at gene TSS, with stronger depletion at upregulated genes (*green*) when compared to downregulated (*orange*) or not differentially expressed (NDE, *grey*) genes. Each inset panel label indicates the RNA-seq dataset from which each gene subset was analyzed. *Right panels*, Quantification of the local minimum for each condition within each inset graph from ChIP-seq profiles. Source data

To investigate molecular mechanisms whereby histone H1.0 participates in fibroblast activation, we used RNA-seq to determine the transcriptome changes after TGF-β treatment that are dependent on histone H1.0. Depletion of histone H1.0 prevented a select subset of transcriptional changes induced by TGF-β treatment (Fig. 3a and Extended Data Fig. 5a). Ingenuity Pathway Analysis (IPA) revealed that upregulated genes whose expression was blocked by histone H1.0 knockdown are involved in key intracellular and extracellular processes (Fig. 3b). KEGG analyses revealed an enrichment in pathways associated with ECM (Fig. 3c and Extended Data Fig. 5c) and signaling via protein kinase B/Akt (activation of which is histone H1.0 dependent (Extended Data Fig. 5c)), a protein associated with growth and proliferation. Among histone H1.0 target genes was *Thbs4* (thrombospondin 4), a secreted ECM protein known to positively regulate tissue healing and previously shown to be necessary for normal fibrotic deposition after cardiac muscle injury^29^. Knockdown of histone H1.0 blocked the TGF-β-induced increase in THBS4 at the transcript and protein level (Extended Data Fig. 5d). Thbs4 was required for TGF-β-induced activation of myofibroblast genes periostin and αSMA expression (Extended Data Fig. 5e,f), demonstrating this to be a necessary downstream gene regulatory target of histone H1.0.

**Fig. 4:**
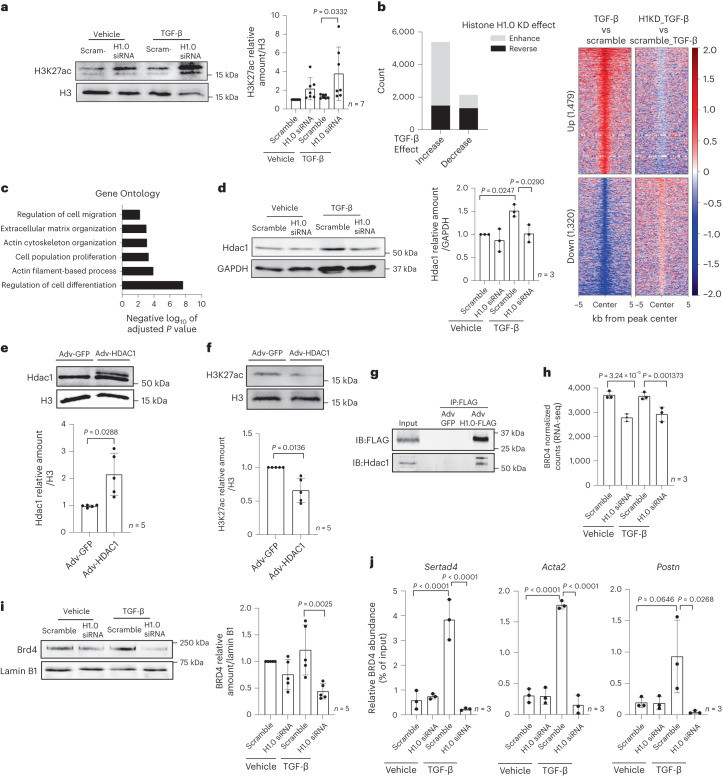
Histone H1.0 depletion prevents TGF-β-induced histone H3K27Ac and modulates actions of HDAC1 and BRD4. **a**, Immunoblot showing changes in global H3K27Ac after histone H1.0 depletion (left) and quantification (right) (mean ± s.d., one-way ANOVA with a post hoc Tukey test; one replicate is a single isolation followed by treatment as indicated and western blotting). **b**, Left: stacked bar chart showing direction of histone H1.0 KD-induced H3K27ac occupancy change (H1.0 KD + TGF-β relative to TGF-β treatment alone, *y* axis) in regions undergoing significant (FDR < 0.05) change in H3K27ac with TGF-β alone. Black and gray coloring indicate regions undergoing reversed or enhanced H3K27ac occupancy in the knockdown condition, respectively. Right: heatmap depicting the log_2_fold change in H3K27ac in regions that undergo a significant increase in H3K27ac occupancy with TGF-β and are prevented with histone H1.0 KD (top). A visualization for the opposite behavior: regions with decreased H3K27ac occupancy that is prevented by histone H1.0 KD (bottom). **c**, Gene Ontology analysis of 300 unique genes closest to the regions whose increases in H3K27ac occupancy and increases in transcription after TGF-β are histone H1.0 dependent (Fisher’s one-tailed exact test, corrected by the g:SCS algorithm and multiple comparison adjustment). **d**, Western blot of HDAC1 showing the effect of histone H1.0 KD on TGF-β-induced Hdac1 upregulation (left) and quantification (right; mean ± s.d., one-way ANOVA with a post hoc Tukey test). **e**, Fibroblasts were transfected with human Adv-HDAC1 or Adv-GFP for 48 h, and Hdac1 protein levels were detected by western blot (top) and quantified (bottom; mean ± s.d., Welch’s unpaired *t*-test). **f**, Western blot showing changes in H3K27ac abundance after Hdac1 overexpression (top) and quantification (bottom; mean ± s.d., Welch’s unpaired *t*-test). **g**, Co-IP assay, performed with anti-FLAG antibody using lysates from Adv-GFP or Adv-GFP-H1.0-FLAG transfected fibroblasts, confirms histone H1.0 interaction with Hdac1 (representative of five independent co-IP experiments). **h**, Effect of histone H1.0 knockdown on BRD4 transcript levels (RNA-seq counts with Benjamini–Hochberg-adjusted Wald test *P* value from the DESeq2 package). **i**, BRD4 protein (mean ± s.d., one-way ANOVA with a post hoc Tukey test). **j**, ChIP–qPCR against BRD4 in primary fibroblasts examining TGF-β-induced changes in BRD4 occupancy at the promoters of *Sertad4*, *Acta2* and *Postn* and the effects of histone H1.0 depletion (mean ± s.d., one-way ANOVA post hoc Tukey test). KD, knockdown. Source data

We reasoned that histone H1.0’s ability to directly bind chromatin and alter gene expression underpins these changes in gene expression and fibroblast phenotype. We therefore examined the influence of histone H1.0 levels on RNA polymerase II (RNAP II) expression and activation. Histone H1.0 knockdown decreased the transcript levels of RNAP II subunit A and prevented the TGF-β-induced increase in subunit C, whereas subunit B was slightly increased and subunit D was unchanged by histone H1.0 depletion (Extended Data Fig. 5g,h). Histone H1.0 depletion also decreased the proportion of RNAP II that is serine 2 phosphorylated in response to TGF-β stimulation (Fig. 3d), indicating that histone H1.0 participates in TGF-β-induced activation of transcription in part by regulating RNAP II subunit levels as well as post-translational modification. One mechanism of chromatin remodeling is via histone acetylation, which alters local chromatin compaction and serves to recruit reader proteins, which in turn facilitate engagement of transcriptional machinery^30^. Inhibition of HDACs, which remove acetyl groups from lysines on histones and other proteins, is sufficient to block fibroblast activation^31^. Depletion of histone H1.0 led to an increase in total H3K27Ac (Fig. 4a), a mark associated with transcriptionally active enhancers, suggesting a shift toward more active chromatin. To examine the role of histone H1.0 in regulating chromatin accessibility via acetylation, we conducted histone H3K27Ac chromatin immunoprecipitation followed by sequencing (ChIP-seq) in TGF-β-treated cells in the presence and absence of histone H1.0. Remarkably, depletion of histone H1.0 blocks the locus-specific changes in H3K27Ac induced by TGF-β (Fig. 4b). Genes whose increase in H3K27Ac occupancy was blocked by histone H1.0 knockdown were enriched in pathways associated with cell migration, proliferation and ECM production (Fig. 4c), demonstrating that histone H1.0 is necessary for the proper acetylation of chromatin around these genes.

Increased pressure in the heart, which leads to fibroblast activation, pathologic muscle growth and heart failure, is associated with elevated HDAC activity^32^, and our data demonstrate that histone H1.0 is necessary for TGF-β-induced upregulation of HDAC1 (Fig. 4d). HDAC1 is preferentially expressed in fibroblasts versus muscle cells in the heart^33^, and overexpression of HDAC1 is sufficient to diminish global levels of H3K27Ac (Fig. 4e,f; HDAC inhibition was previously shown to block TGF-β-induced production of ECM^34^). Co-immunoprecipitation (co-IP) demonstrates that histone H1.0 can bind HDAC1 in fibroblasts (Fig. 4g), as previously documented for other HDAC isoforms in human cell lines^35^, implying that this regulatory interaction can also occur at the protein level. Thus, one mechanism by which histone H1.0 can regulate transcription is by preventing upregulation of HDAC and thereby reducing its gene silencing effect.

To further investigate the molecular basis for how altered histone acetylation levels may influence transcription, we examined expression of the chromatin reader protein BRD4, which binds acetylated lysines, facilitating recruitment of positive transcription elongation factor b (P-TEFb), thereby releasing transcriptional pausing. Inhibition of BRD4 was previously shown to block cell growth^36^ and fibroblast activation^37^. We observe that histone H1.0 depletion leads to a decrease in BRD4 transcript and protein levels (Fig. 4h,i), suggesting that reduction in the abundance of this chromatin reader is part of the mechanism by which histone H1.0 inhibition prevents fibroblast activation. These observations are in agreement with histone H1.0 dependence of changes in phosphorylation of RNA Pol II at Ser2 (Fig. 3d), given that BRD4 is known to recruit the essential RNA Pol II regulatory factor P-TEFb^38^ and promote Ser2 phosphorylation^39^. We examined binding of BRD4 to known TGF-β target genes using ChIP–qPCR: TGF-β induced robust recruitment of BRD4 to the transcription start sites (TSSs) of *Sertad4* (ref. ^40^), *Acta2* and *Postn*, which was completely prevented in all cases by depletion of histone H1.0 (Fig. 4j). These findings, together with previous studies, indicate that histone H1.0 coordinates reorganization of H3K27Ac around TGF-β target genes in part by regulating histone acetylation via HDACs and transcription by BRD4.

### Histone H1.0 regulates global chromatin compaction

**Fig. 5:**
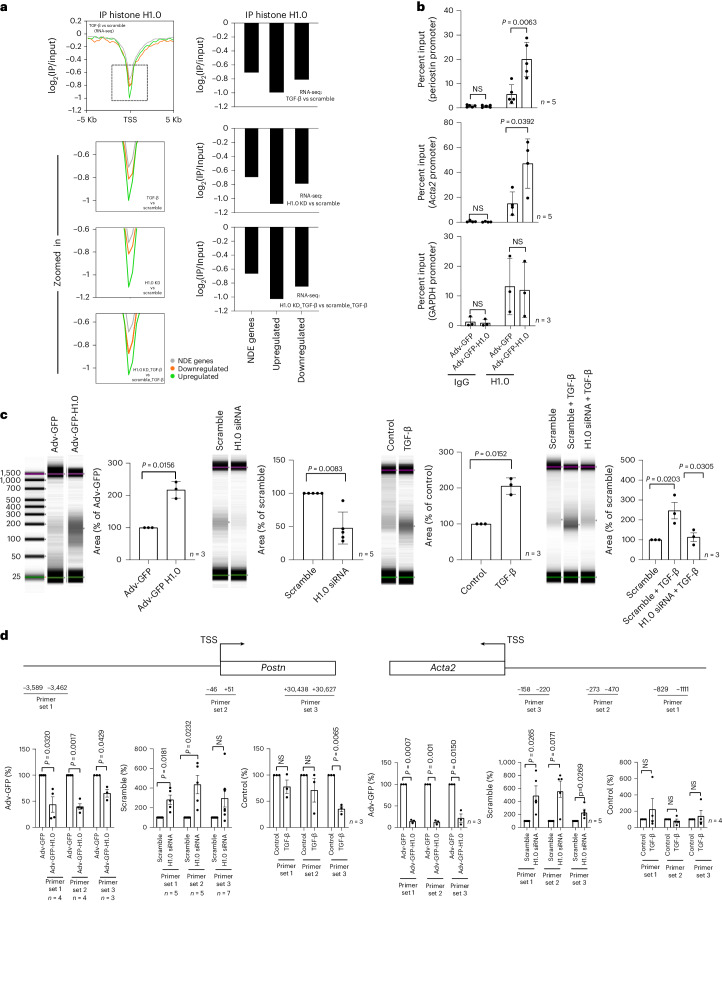
Histone H1.0 levels control chromatin fiber compaction. **a**, Left: ChIP-seq examining histone H1.0 occupancy at TSSs relative to other genomic regions (*y* axis indicates log_2_(IP/input) signal) at differentially transcribed genes: upregulated (green), downregulated (orange) or not differentially expressed (NDE; gray). Labeling in bottom right of inset panel indicates the RNA-seq dataset comparisons. Right: quantification of the local minimum for each condition within each inset graph from ChIP-seq profiles on the left. **b**, ChIP–qPCR performed in isolated murine cardiac fibroblasts transfected with Adv-GFP (control) or Adv-GFP-H1.0 examining histone H1.0 occupancy in the promoter region of *Periostin* (top), *Acta2* (middle) and *Gapdh* (bottom) (mean ± s.d., Welch’s unpaired *t*-test; one replicate is a single isolation followed by treatment as indicated and qPCR). **c**, Gel images of nuclease-digested genomic DNA (wherein more compact chromatin will experience less digestion and migrate higher on the gel) from fibroblasts transfected with Adv-GFP-H1.0 or Adv-GFP control (first panel), histone H1.0 siRNA or scrambled siRNA control (second panel), treated with TGF-β (third panel) or treated with TGF-β in the presence or absence of histone H1.0 (fourth panel). Quantification of genomic DNA in the 100–300-bp range is shown next to each gel image (mean ± s.d., one-way ANOVA with a post hoc Tukey test; one replicate is a single isolation followed by treatment as indicated and gel densitometry). **d**, Top: location of primers used for qPCR on DNA from fibroblasts after histone H1.0 overexpression or knockdown before TGF-β treatment. Bottom: qPCR measured amount of DNA (less signal indicates less DNA and, thus, greater compaction of region in question; mean ± s.e.m., Welch’s unpaired *t*-test; one replicate is a single isolation followed by treatment as indicated, MNase digestion and qPCR). KD, knockdown; NS, not significant. Source data

Unlike transcription factors or some modified core nucleosome histones, the distribution of linker histone H1 across the genome is fairly ubiquitous^17,41^. We performed ChIP-seq for histone H1.0 (Extended Data Fig. 5b) and investigated regions of relative depletion as described^42^. Histone H1.0 is depleted at genes undergoing altered expression after fibroblast activation, with a greater depletion observed in genes whose expression is increased (Fig. 5a), suggesting that histone H1.0 eviction is associated with chromatin relaxation^18^. We confirmed specific localization of histone H1.0 to several TGF-β target genes using ChIP–qPCR (Fig. 5b), indicating that, although its genomic distribution is broad, it is not uniform. Immunoprecipitation of endogenous histone H1.0 (Fig. 5a) or overexpressed, tagged histone H1.0 (Extended Data Fig. 5i) gave similar results. We next directly tested the role of histone H1.0 in chromatin folding by performing nuclease digestion of genomic DNA to reveal the relative ratio of compact (nuclease inaccessible) to relaxed (nuclease accessible) DNA. Overexpression of histone H1.0 increased the proportion of DNA that was compacted and, thus, nuclease inaccessible, whereas knockdown of histone H1.0 had the antithetical effect (Fig. 5c). Interestingly, treatment with TGF-β shifted the genome to a more compact state, and knockdown of histone H1.0 reversed this effect (Fig. 5c), demonstrating that this behavior of histone H1.0 to modulate chromatin fiber accessibility is operative in the context of fibroblast activation. We performed targeted PCR for regions of *Acta2* and *Postn*, demonstrating that the presence of histone H1.0 tended to compact these regions of chromatin (Fig. 5d). Histone H1.0 overexpression or TGF-β treatment renders the *Acta2* and *Postn* loci less accessible (thus, less DNA was recovered by PCR), whereas knockdown of histone H1.0 has the opposite effect. These findings suggest that normal levels of histone H1.0 establish a microenvironment for expression or repression of genes, such that perturbing the balance of histone H1.0 levels prevents normal stress-activated transcription.

**Fig. 6:**
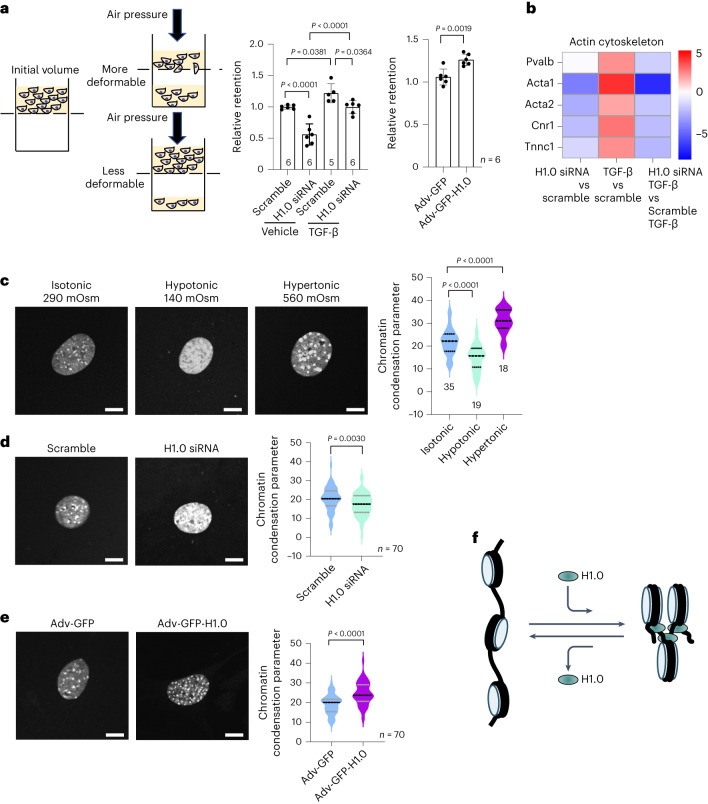
Histone H1.0 levels directly influence cellular stiffness and nuclear condensation. **a**, Left: diagram of the cellular filtration assay. Right: effect of histone H1.0 depletion and TGF-β on cellular retention. Two-minute applied pressure for KD and TGF-β; for H1.0 overexpressing cells, more than 4 min were required because of increased stiffness (mean ± s.d., one-way ANOVA with a post hoc Tukey test (knockdown and TGF-β groups) or Welch’s *t*-test (H1.0 overexpression); one replicate is a single isolation followed by treatment as indicated and cellular filtration measurement). **b**, Heatmap depicting actin cytoskeleton genes and their expression after TGF-β in the presence or absence of histone H1.0. **c**, Left: DAPI staining of primary fibroblasts incubated under different osmotic environments for 1 h. Right: CCP quantifies decondensation induced by hypotonic conditions and condensation induced by hypertonic conditions. **d**, Left: DAPI staining of primary fibroblasts depleted of histone H1.0. Right: CCP, same as in **c** (*n* = 70 nuclei per group). **e**, Left: DAPI staining of primary fibroblasts transfected with Adv-GFP-H1.0 or Adv-GFP control. Right: CCP, same as in **c** (*n* = 70 nuclei per group). Violin plots indicate mean and quartiles for **c**–**e** (one-way ANOVA with post hoc Tukey test, representative of three biological replicates). Scale bars, 10 μm. **f**, Schematic illustration of the effect of histone H1.0 abundance on chromatin compaction. Source data

**Extended Data Fig. 6:**
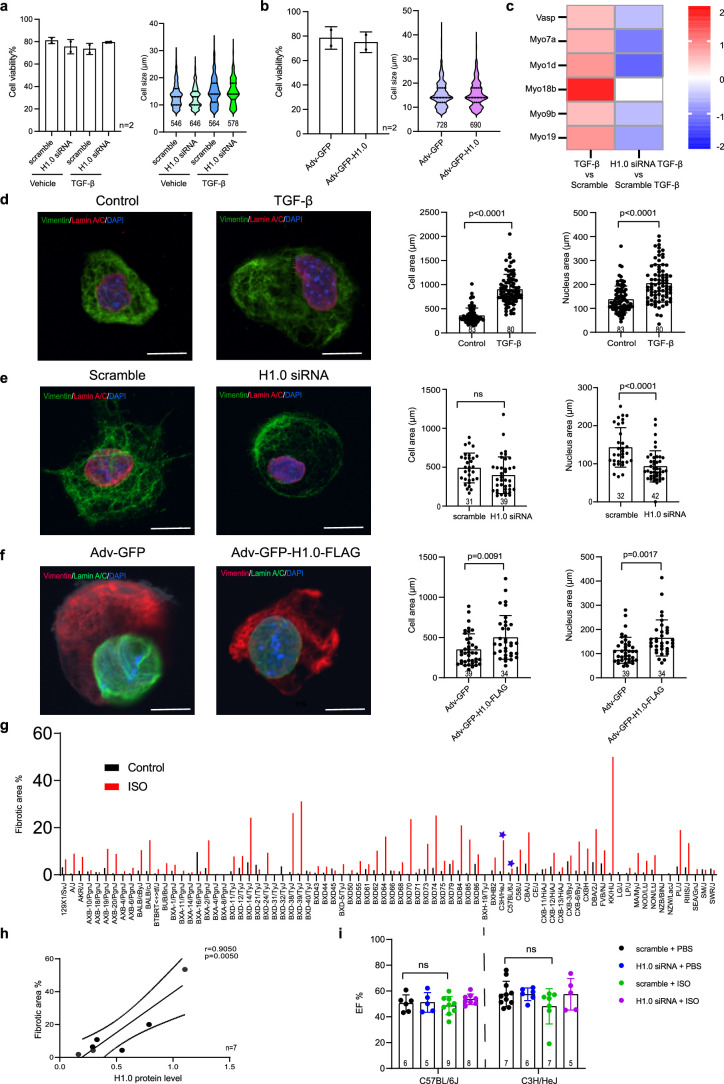
Modulation of histone H1.0 levels does not affect fibroblast viability or size. **a**) Cell viability (*left*) and cell size (*right*) analyses of isolated mouse cardiac fibroblasts transfected with histone H1.0 siRNA or scrambled negative control and treated with vehicle or TGF-β show no changes among groups (representative of 2 biological replicates; mean ± SD plotted for cell viability, median and quartiles indicated for cell size). **b**) Cell viability (*left*) and cell size (*right*) analyses of fibroblasts transfected with Adv-GFP-H1.0 or Adv-GFP control show no changes among groups (representative of 2 biological replicates; mean ± SD plotted for cell viability, median and quartiles indicated for cell size). **c**) Heatmap depicting several myosin and focal adhesin genes whose increased expression after TGF-β is prevented by histone H1.0 knockdown. **d**) *Left*, Fibroblasts treated with vehicle or TGF-β were immunolabeled with anti-Vimentin (*green*) and anti-Lamin A/C (*red*) antibodies (*left*). DAPI (*blue*) stains cell nuclei. Scale bar=10μm. *Right*, Cell and nuclear area quantification (mean ± SD, Welch’s unpaired t-test). **e**) *Left*, Fibroblasts transfected with histone H1.0 siRNA or scrambled negative control were immunolabeled with anti-Vimentin (*green*) and anti-Lamin A/C (*red*) antibodies. DAPI (*blue*) stains cell nuclei. Scale bar=10μm. Histone H1.0 KD promoted a reduction in nuclear area without altering cell size. *Right*, Cell and nuclear area quantification (mean ± SD, Welch’s unpaired t-test). **f**) *Left*, Fibroblasts transfected with Adv-GFP-H1.0 or Adv-GFP control were immunolabeled with anti-Vimentin (*red*) and Lamin A/C (*green*) antibodies. DAPI (*blue*) stains cell nuclei. Scale bar=10μm. Histone H1.0 overexpression resulted in increased cell and nuclear size. *Right*, Cell and nuclear area quantification (mean ± SD, Welch’s unpaired t-test). **g**) Examination of fibrotic area in genetically distinct mouse strains undergoing isoproterenol treatment (Rau et al. Circ CV Genetics, 2015); asterisks indicate strains used in this study. **h**) Pearson’s correlation between histone H1.0 protein level and fibrotic area in hearts treated with ISO in the presence or absence of histone H1.0 depletion by siRNA. **i)** Neither ISO nor histone H1.0 KD affected ventricular ejection fraction in C57BL/6 J and C3H/HeJ mice (mean ± SD, one-way ANOVA with a post-hoc Tukey test within strains). Source data

Nuclear deformability has been implicated in diseases such as cancer and fibrosis^5^ and is a major contributor to whole cell rigidity^43^. We therefore sought to investigate a role for histone H1.0 to control global genome and nuclear stiffness by employing a deformability assay^44^. Depletion of histone H1.0 had a robust effect to increase the deformability of cells under basal conditions and, to a lesser degree, after stimulation with TGF-β (Fig. 6a). Neither cell viability nor cell size was significantly altered by modulating histone H1.0 levels (Extended Data Fig. 6a,b), whereas TGF-β treatment increased the size and stiffness of cells independent of the nucleus (Extended Data Fig. 6d–f), likely contributing to the muted effect of histone H1.0 depletion on cellular deformability measurements after TGF-β. In contrast, increasing the abundance of nuclear histone H1.0 was sufficient to increase cellular retention in the absence of TGF-β (Fig. 6a), which is consistent with increased cell and nuclear stiffness. Upregulation of numerous cytoskeletal genes by TGF-β was blocked by depletion of histone H1.0 (Fig. 6b; myosins were also under control of histone H1.0; Extended Data Fig. 6c), providing a mechanistic explanation for the effect of histone H1.0 depletion to alter cell compliance changes after TGF-β. To directly evaluate the role of histone H1.0 in genome compaction, we imaged nuclei and quantified the chromatin condensation parameter (CCP), a measurement of global chromatin architecture^45^, after modulation of histone H1.0 levels or hypotonic or hypertonic treatments as positive controls (which, respectively, decompact or compact chromatin; Fig. 6c). Depletion of histone H1.0 levels caused global chromatin decondensation (Fig. 6d), whereas augmentation of histone H1.0 caused condensation (Fig. 6e). Combined with Fig. 5, these findings demonstrate that histone H1.0 modulates chromatin compaction on a genome-wide scale—directly controlling overall cell deformability—via a local mechanism in which more histone H1.0 leads to more restrictive topology (Fig. 6f).

### Histone H1.0 controls fibrosis in vivo

**Fig. 7:**
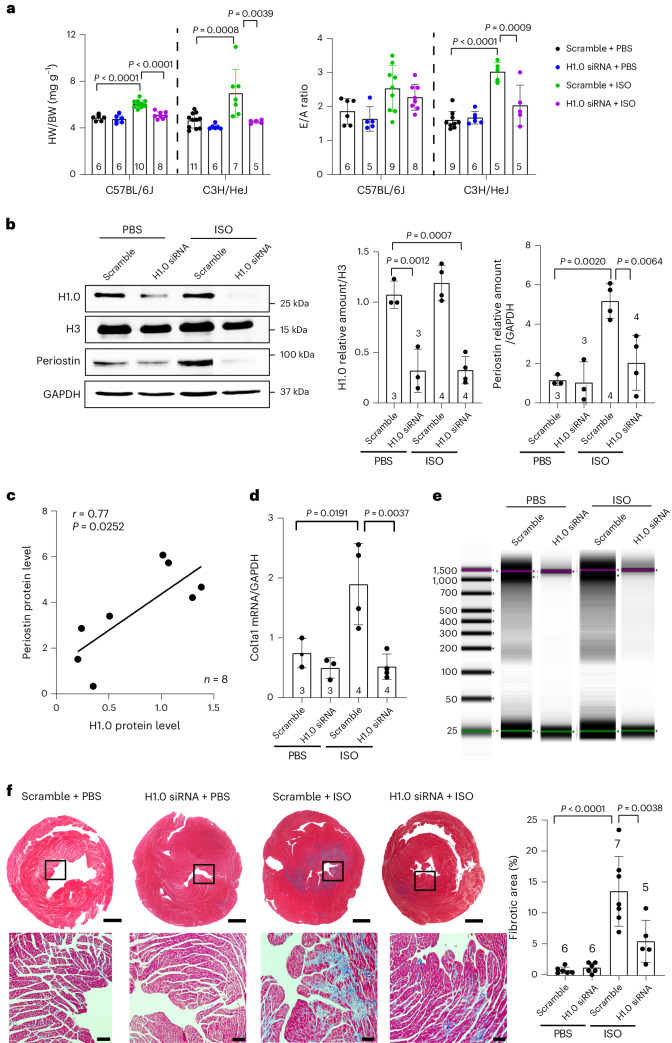
Histone H1.0 depletion prevents disease-associated cardiac fibrosis in vivo*.* **a**, Left: heart weight (HW) to body weight (BW) ratios. Right: E/A ratios. **b**, Western blot from whole heart showing histone H1.0 KD effect on ISO-induced periostin activation and quantification. **c**, A positive Pearson’s correlation was observed between histone H1.0 and periostin levels in hearts from C57BL/6J mice. **d**, RT–qPCR showing effect of histone H1.0 KD on ISO-induced changes in *Col1a1* transcript abundance. **e**, Gel images of genomic DNA digested from hearts of C57BL/6J mice. **f**, Left: Masson’s trichrome staining of heart sections to measure fibrosis (C3H/HeJ mice; bars in whole heart images, 1 mm; bars in zoomed images, 100 μm; square indicates the region from which high-magnification images were acquired). Right: quantification of fibrotic area. All data are presented as mean ± s.d., analyzed by one-way ANOVA with post hoc Tukey test. Source data

**Extended Data Fig. 7:**
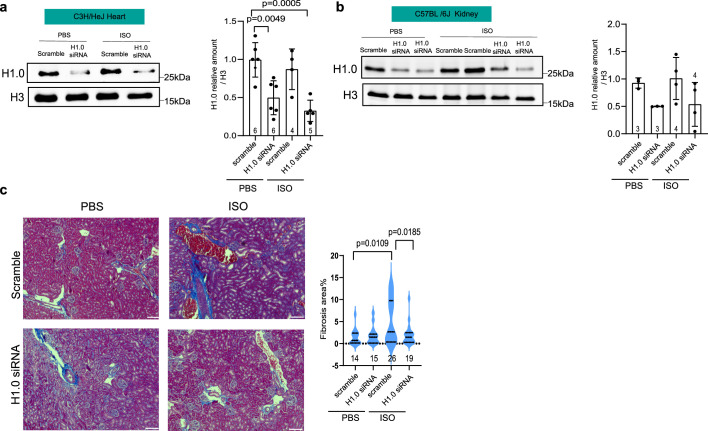
Depletion of histone H1.0 prevents fibrosis *in vivo.* **a)** *Left*, Efficiency of histone H1.0 KD was examined by Western blot in hearts of C3H/HeJ mice. *Right*, Immunoblot quantification (mean ± SD, one-way ANOVA with post-hoc Tukey test). **b)** *Left*, Western blot shows histone H1.0 protein levels from C57BL/6 J kidney after ISO treatment and depletion of histone H1.0. *Right*, Immunoblot quantification (mean ± SD one-way ANOVA with a post-hoc Tukey test). **c)** *Left*, Representative Masson trichrome staining of C57BL/6 J kidney, showing that histone H1.0 KD reduces kidney fibrosis in the ISO-treated group. Scale bars: 100 µm. *Right*, Image quantification (median and quartiles shown; one-way ANOVA with post-hoc Tukey test; n ≥ 5 sections/group, ≥14 images per group as indicated in figure). Source data

**Extended Data Fig. 8:**
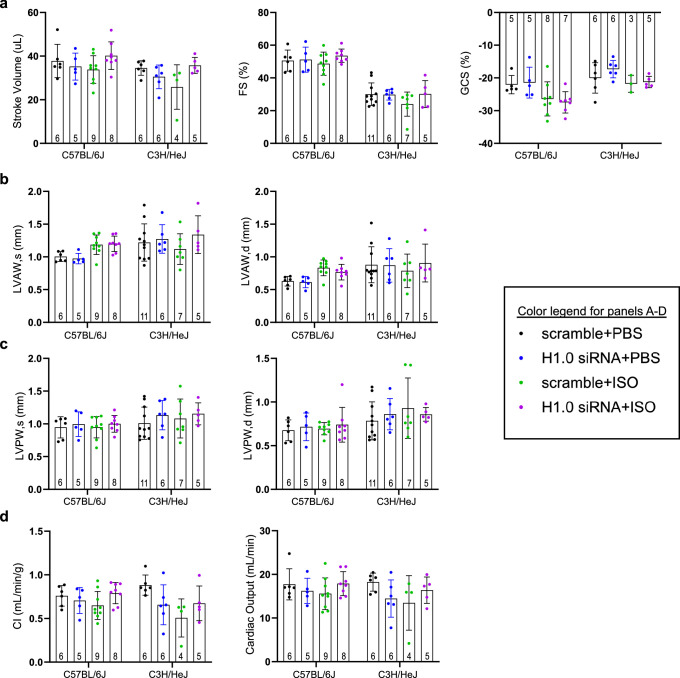
Echocardiography measurements after *in vivo* histone H1.0 knockdown and ISO treatment. **a**) Mice from both C57BL/6 J and C3H/HeJ backgrounds were subjected to tail vein injection of scramble siRNA or siRNA against histone H1.0, concurrent with PBS or isoproterenol treatment. Echocardiography was performed to measure stroke volume (left), fractional shortening (FS, middle), and global circumferential strain (GCS, right). **b**) Left ventricular anterior wall thickness (LVAW) in systole (left) and diastole (right). **c**) Left ventricular posterior wall thickness (LVPW) in systole (left) and diastole (right). **d**) Cardiac index (CI, left), and cardiac output (right). Mean ± SD displayed in all panels; no significant differences were observed. Source data

**Extended Data Fig. 9:**
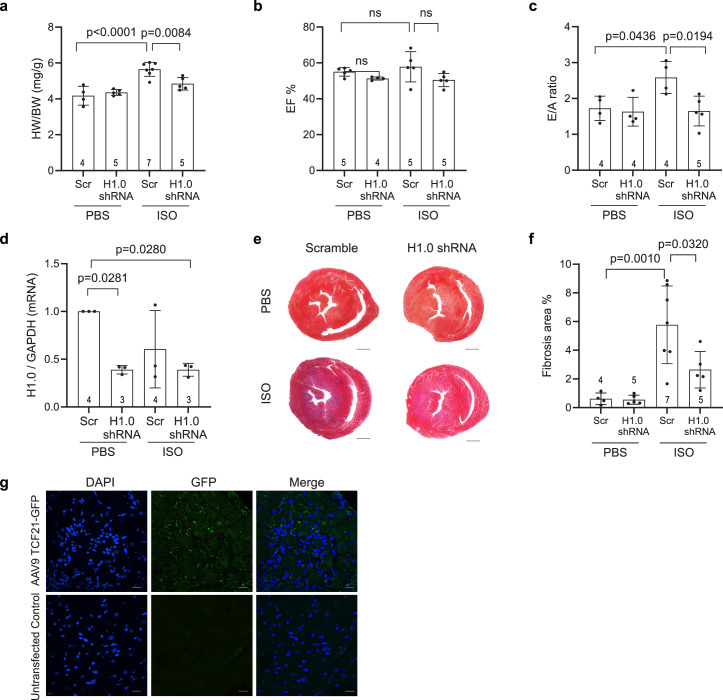
AAV9 delivery of shRNA *in vivo* depletes histone H1.0 and prevents isoproterenol-induced fibrosis. **a**) *In vivo* histone H1.0 knockdown attenuates ISO-induced cardiac hypertrophy as measured by heart weight to body weight ratio (HW/BW) after an AAV9-based *in vivo* knockdown approach targeting shRNA against histone H1.0 (AAV9-Tcf21-GFP-shRNAmir-H1.0 shRNA or AAV9-CMV-GFP-shRNAmir-scramble shRNA). The AAV9 is taken up by fibroblasts and myocytes in heart: the fibroblast-specific Tcf21 promoter drives GFP and the shRNAmir promoter drives transcription of the shRNA. This strategy allows us to identify fibroblasts taking up the vector (they will express GFP, myocytes will not) and in those cells characterize the level of histone H1.0 depletion. 3×10^11^ GC were injected. In the control vector, GFP is driven by CMV promoter and scramble shRNA (Scr) is driven by U6. 5 weeks post AAV9 injection, PBS or ISO (20 mg/kg/day) were injected for one week. Physiological endpoints were then examined via gross histology, ECHO, and PCR (n ≥ 4/group). **b**) Ejection fraction (EF) is not significantly changed after *in vivo* shRNA injection and/or one week ISO treatment. **c**) Histone H1.0 depletion attenuates ISO-induced diastolic dysfunction as measured by E/A ratio (n ≥ 4/group). **d**) Quantification of histone H1.0 depletion by RT-qPCR (n ≥ 3). **e**) Representative images from of Masson’s trichrome staining to examine cardiac fibrosis after *in vivo* histone H1.0 knockdown and/or ISO treatment (n ≥ 4/group). **f**) Quantitation of fibrotic area shows that depletion of histone H1.0 significantly inhibits ISO-induced fibrosis. Mean ± SD, one-way ANOVA with post-hoc Tukey test for panels (**a-d** and **f**). **g**) Antibody-based detection of GFP in hearts of animals receiving AAV9 or untransfected controls. Scale bar = 10 µm (representative of n = 3 biological replicates). Source data

To investigate a role for histone H1.0 to control responses to physical stress in vivo, we employed a model of catecholamine stimulation with ISO, a non-selective β-adrenergic receptor agonist that increases cardiac work and, thus, tension on the muscle fiber, in addition to inducing fibrosis^46^. Notably, catecholamines can also directly increase cellular tension in non-muscle cells^47,48^. Previous studies showed that the fibrotic effects of ISO are a complex trait strongly influenced by the genetic background of the mouse^22^, and thus we chose two strains of mice to examine: C57BL/6J, which has a modest fibrotic response, and C3H/HeJ, which exhibits a more pronounced response (Extended Data Fig. 6g). Our analyses of C3H/HeJ mice demonstrated a positive correlation between histone H1.0 abundance and fibrotic deposition after ISO treatment (Extended Data Fig. 6h). Administration of ISO induced cardiac muscle hypertrophy in both strains, which was attenuated by co-administration of siRNA against histone H1.0 (Fig. 7a,b). Furthermore, in vivo depletion of histone H1.0 blocked ISO from inducing an increase in the ratio of early to late peak diastolic filling velocities (E/A ratio) (Fig. 7a,b), a measure of diastolic function, where an E/A ratio ≥2 is consistent with a less compliant, stiffer ventricle. No effect on ejection fraction, a measurement of systolic function, was observed after histone H1.0 depletion (Extended Data Figs. 6i and 8). siRNA treatment was sufficient to deplete H1.0 in both mouse strains (Fig. 7b and Extended Data Fig. 7a) as well as to block activation of fibrotic genes, including periostin and collagen 1A1, as measured by protein and transcript abundance (Fig. 7c,d). Knockdown of histone H1.0 was sufficient to transiently decondense chromatin in heart muscle as measured by nuclease accessibility (Fig. 7e), indicating that the in vivo mechanisms of protection work through actions of histone H1.0 to globally remodel the genome through actions at the chromatin fiber. We observed a significant prevention of ISO-induced fibrosis (Fig. 7f), demonstrating that histone H1.0 is essential for the transcriptional program driving production of ECM in vivo. We also found fibrosis in the kidneys of these ISO-treated animals, which was partially attenuated by depletion of histone H1.0 (Extended Data Fig. 7b,c). Notably, in vivo depletion of histone H1.0 with an orthogonal technique (AAV9-mediated delivery of shRNA against a different region of the histone H1.0 transcript) was sufficient to recapitulate the phenotypes observed with siRNA-mediated depletion (Extended Data Fig. 9). Taken together, these findings demonstrate a powerful effect of histone H1.0 to regulate fibrosis in vivo through its actions to control chromatin packaging.

## Discussion

One manner in which cells alter their microenvironment in response to physical and chemical stressors is via fibrosis, or the deposition of ECM proteins, thereby altering parenchymal mechanics. We observe that tuning of histone H1 levels and chromatin compaction is necessary for response to stress stimuli and that the linker histone H1.0 isoform has a privileged role in this process. We also demonstrate that augmenting histone H1.0 levels can recapitulate the chromatin organization, gene expression and mechanical cell behaviors in the absence of changes to cellular tension or cytokine stimulation. These findings support a central role for histone H1.0 as a molecular regulator of fibroblast stress response, coupling chromatin organization with cellular mechanical properties.

Alteration of histone H1 levels in vivo has been shown to shift global chromatin architecture between a relaxed (less H1) or more compact (more H1) state, and depletion of H1 promotes the development of lymphoma^18^, indicating that remodeling genome packaging through histone H1 is a conserved mechanism across cell types. Our findings indicate that the levels of histone H1.0 can control chromatin condensation and thereby expression of genes associated with the cytoskeleton, force generation, ECM and cellular motility. These findings raise the intriguing possibility that histone H1.0 may work directly via changes in nuclear compliance—that is, to change nuclear stiffness as a mechanism to change cell stiffness—in parallel with effects of histone H1.0 to control transcription of genes associated with altering cellular rigidity. We demonstrate that the abundance of histone H1.0 on chromatin is directly associated with chromatin compaction: depleting histone H1.0 led to fiber relaxation and global decondensation, whereas overexpression had the opposite effect. These changes in chromatin accessibility prime the actions of other chromatin remodelers, such as HDAC1 and BRD4, which, in turn, modulate transcription of stress-responsive genes in a histone H1.0-dependent manner. We speculate that the observed role of histone H1.0 to influence TGF-β-induced Pol2 phosphorylation may be dependent on the concomitant effects on BRD4 and H3K27 acetylation in the context of transcriptional regulation.

The enrichment of histone H1.0 that we observed across fibroblast populations is in agreement with this isoform being the only polyadenylated version of the linker histone family^49^ and, thus, the only one likely to be strongly expressed in non-dividing cells. Deletion of histone H1.0 in vivo did not adversely affect mouse development^15^, likely due to compensation by other linker histone family members (linker-to-core nucleosome ratio was unchanged in these animals^15^). Subsequent studies depleted other histone H1 isoforms by germline knockout: loss of individual isoforms H1.3, H1.4 or H1.5 failed to influence mouse development—including when combined with simultaneous loss of histone H1.0 in a double knockout model—again due to compensatory upregulation of other linker histone isoforms^14^. Triple knockouts for H1.3, H1.4 and H1.5 were lethal, with no embryos surviving past embryonic day (E) 11.5 (ref. ^16^). Although the exact stoichiometry of linker histones to nucleosome core particles at individual loci is uncertain and likely varies across the genome (and between different cell types), in somatic cells the average ratio approximates 1. When the linker-to-core ratio is maintained around 1, by altered expression of other isoforms in the setting of genetic knockout, there is no overt phenotype, whereas, in the setting of triple deletions, a decreased linker-to-core ratio is associated with widespread developmental defects^16^. In the present study, transient knockdown of histone H1.0 was associated with neither major alterations in other linker histones nor changes in the expression of the core nucleosome histones (H2A, H3 and H4, with minimal change in H2B); thus, our intervention induced a transient decrease in the linker-to-core histone ratio, concomitant with perturbations in chromatin structure and the responsiveness of the cells to growth stimulus. Analysis of single-nucleus RNA-seq data from dilated and arrhythmogenic cardiomyopathies^50^ revealed that histone H1.0 was the only variant increased in fibroblast subpopulations that also expressed periostin, and other scRNA-seq datasets from human hearts all show a positive correlation between histone H1.0 and periostin (Extended Data Fig. 1). Previous work showed that levels of histone H1 variants are dynamic during development and reprogramming in the mouse embryo^51^ as well as in the adult mouse heart after pressure overload^25^, linking changes in global chromatin organization with the phenotypic shifts associated with maturation and disease.

Our results support a model in which perturbation of normal chromatin architecture is an organizing feature to regulate cellular response to stress. This mechanism is centrally controlled by levels of histone H1.0 and is used by the cell to alter local chromatin compaction and to change the global stiffness of the cell to respond to altered mechanical or cytokine environment. Recent investigations have shown that linker histones participate in gene regulation through mechanisms beyond their ability to compact the chromatin fiber^52^. The actions of histone H1.0 are not merely to turn genes on or off. We found that chromatin fibers are relaxed by histone H1.0 depletion, in agreement with previous studies of linker histones, yet histone H1.0 levels alone are not predictive of transcription. For example, the stress-activated genes periostin and αSMA, whose increase in expression is blocked by histone H1.0 depletion, are bound by histone H1.0 and more compacted when it is present. Thus, during agonist stimulation, histone H1.0-dependent changes in chromatin organization must facilitate the actions of other transcriptional machinery.

At the molecular level, the actions of histone H1.0 in cardiac fibroblasts involve direct binding to chromatin and compacting local fibers. Our data demonstrate that histone H1.0 levels influence global and gene-specific deposition of histone H3K27Ac, a mark of gene and enhancer activation. Depletion of histone H1.0 completely blocked changes in H3K27Ac induced by TGF-β, including in fibrosis-associated genes, notwithstanding global changes in acetylation being increased after histone H1.0 knockdown, demonstrating the specificity of this regulation. This process also involved decreased expression of HDAC1 and decreased expression of BRD4, a bromodomain containing histone reader necessary for binding to acetylated histones to recruit transcriptional machinery. Small-molecule-based HDAC inhibition can prevent fibrosis in the heart^53–55^. Similarly, small-molecule-based inhibition of BRD4 has independently been shown to block fibrosis in the lung^56^, liver^57^ and heart^40,58^. In addition, previous studies in fibroblasts showed that HDAC inhibition blocks BRD4-dependent gene activation^31^. Depletion of histone H1.0 led to a decrease in HDAC levels and a global decondensation of chromatin, in agreement with previous work showing that HDAC inhibition caused global chromatin decondensation and deacetylation in living cells^59^ and plays a critical role in genome protection during the mechanical disruptions of mitosis^60^. Histone H1 itself has been shown to be regulated by acetylation and HDAC inhibition—either by direct interaction with H1 or through chromatin decondensation—has been shown to increase histone H1 mobility on chromatin^61^. Both histone acetylation and BRD4 levels have been shown to drive chromatin phase separation^62^, a behavior linked to the global condensation and local fiber compaction. Our findings provide a molecular link between the actions of histone H1.0 to regulate chromatin condensation and the previous observations of these histone modifiers in the setting of pathologic fibroblast activation.

Although the effects on fibrosis were quite similar, we observed variability in the extent of protein and transcript knockdown comparing the siRNA versus AAV9-shRNA approaches, likely reflecting distinct mechanisms of delivery (these approaches also cannot rule out effects in non-fibroblasts). To translate these observations into a therapy, significant optimization would be required to engineer a potent, targeted approach for antisense-based depletion of histone H1.0 in vivo. Although histone H1.0 is dispensable for organismal development due to compensatory upregulation of other isoforms, our findings reveal a necessary role for this protein to regulate fibroblast activation and cellular stiffness in the adult mouse. Notably, knockdown of histone H1.0 alone had discernable effects neither on cell physiology in culture nor on organ function or histology in vivo. These observations likely represent a distinct behavior of chromatin in non-proliferating adult cells: in response to stress, global chromatin changes are necessary for transcriptional activation and involve histone H1.0, mirroring distinct effects shown for other chromatin structural proteins, such as CTCF in primordial versus terminally differentiated cells^63,64^. These changes in chromatin architecture are responsive to cytokines such as TGF-β and hormones such as angiotensin II and ISO, serving to directly alter cellular stiffness by changing nuclear deformability and by ensuring proper expression of cytoskeletal and ECM proteins. In this model, histone H1.0 directly links chromatin structure with cellular stress response, providing a mechanism to ensure that the microenvironment is coupled to the necessary transcriptional program to elicit distinct mechanical behaviors of the cell.

## Methods

### Animal care and use

All animal studies were approved by the University of California, Los Angeles (UCLA) Animal Research Committee in compliance with the National Institutes of Health Guide for the Care and Use of Laboratory Animals. Adult female and male C57BL/6J (The Jackson Laboratory, 000664) and C3H/HeJ (The Jackson Laboratory, 000659) mice (8–12 weeks old) were obtained and used in this study. Male and female mice were used in this study, but the groups were not powered to reveal sex differences. Animals were housed under normal light/dark cycles and with controlled ambient air temperature and humidity.

### Histone H1.0 knockdown in vivo in mice

H1.0 siRNA (Thermo Fisher Scientific, 4404010) and scrambled negative control siRNA (Thermo Fisher Scientific, 4457289) were purchased. For each experimental group, siRNA (1.25 mg kg^−1^ body weight) was tail vein injected into either C57BL/6J or C3H/HeJ mice on days 0, 7 and 14 using Invivofectamine 3.0 (IVF3005, Thermo Fisher Scientific) according to the manufacturerʼs instructions and based on previous studies^65^. siRNA sequences are listed in Supplementary Table 2.

### Murine models of fibrosis

Cardiac fibrosis was induced by daily intraperitoneal injection of ISO (MilliporeSigma, 16504) for 14 d (C57BL/6J: 80 mg kg^−1^ day^−1^; C3H/HeJ: 20 mg kg^−1^ day^−1^), beginning the day of the second siRNA injection (day 7 of the in vivo knockdown protocol described above). Mice in negative control groups were injected daily with vehicle (PBS). Mice were euthanized 14 d after ISO treatment and then hearts, kidneys and lungs were harvested.

### Echocardiography

Heart function was measured by echocardiology before and after ISO treatments. Animals were anesthetized with 1.5% isoflurane and 95% O_2_, and chest hair was removed. Continuous ECG monitoring was implemented, and heart rates were maintained between 400 and 500 beats per minute. Body temperature was set at 37 °C using a heating pad. A Vevo 3100 imaging system was used to acquire M-mode images. LV systolic function was evaluated by calculating ejection fraction (EF%). LV diastolic function was measured by calculating the E/A ratio. All calculations were performed using the Vevo LAB 5.6.1 system.

### Cell culture and TGF-β or angiotensin II treatment

Adult female and male C57BL/6J primary cardiac fibroblasts were isolated using enzymatic digestion (7 mg ml^−1^ collagenase type II: Worthington Biochemical, LS004177) followed by centrifugation (644*g* for 8 min at 4 °C) and cell plating in DMEM/F12 media supplemented with 10% FBS, 1% antibiotics (penicillin and streptomycin) and 0.1% insulin-transferrin-selenium (ITS; Corning, 354350). After 2 h, cells were maintained in DMEM/F12 media supplemented with 10% FBS, 1% antibiotics (penicillin and streptomycin), human basic fibroblast growth factor (hbFGF; 1:10,000 concentration from 200× stock, MilliporeSigma, 11123149001) and 0.1% ITS (Corning, 354350). Media and floating cells were then removed, and fibroblasts were grown in DMEM/F12 media supplemented with 10% FBS, 1% antibiotics, hbFGF and 0.1% ITS until reaching 70–80% confluency.

Lung primary fibroblasts were isolated from adult female and male C57BL/6J mice using a previously reported method involving collagenase digestion^66^ and maintained in DMEM/F12 media supplemented with 20% FBS, 1% antibiotics (penicillin and streptomycin), hbFGF and 0.1% ITS until reaching 70–80% confluency. Mouse skin fibroblasts from passages 3–5 and human skin fibroblasts from passage 9 were maintained in DMEM/F12 media supplemented with 10% FBS and 1% antibiotics (penicillin and streptomycin). For all experiments involving fibroblasts, cells at 70–80% confluency were cultured in serum-free media (0.1% FBS) for 24 h before TGF-β treatment (10 ng ml^−1^; Novoprotein, CA59) or angiotensin II treatment (1 µM, Sigma-Aldrich, A9525).

### Immunoblotting and antibodies

Protein was extracted from primary cardiac fibroblasts and lung fibroblasts using homemade RIPA lysis buffer (150 mM NaCl, 5 mM EDTA, 50 mM Tris pH 8.0, 1% NP-40, 0.5% sodium deoxycholate, 0.1% sodium dodecyl sulfate (SDS)) containing protease inhibitors (Roche, 04693159001) and phosphatase inhibitors (Roche, 04906837001). A homemade lysis buffer was used for protein extraction from whole heart, lung or kidney tissue (50 mM Tris pH 7.4, 10 mM EDTA, 1% SDS, 10 mM sodium butyrate, 1.2 mM phenylmethanesulfonyl fluoride, 1 mM sodium fluoride, 1 mM sodium orthovanadate) supplemented with protease inhibitor tablets (Roche, 04693159001). Protein concentration was measured using a Pierce BCA Protein Assay (Thermo Fischer Scientific, 23225). An equal amount of protein was loaded into an SDS-containing polyacrylamide gel. After electrophoresis, proteins were transferred to a nitrocellulose membrane (Bio-Rad, 1620115). Membranes were blocked with 5% BSA for 1 h, incubated with appropriate primary and fluorescent secondary antibodies and developed using a ChemiDoc MP Imaging System (Bio-Rad). All primary and secondary antibodies are listed in Supplementary Table 1. Unless otherwise stated, western blots were performed on whole cell lysate, which would include proteins bound to, as well as within, the cells.

### Immunofluorescence

Cells were fixed at either (1) room temperature for 10 min using 4% paraformaldehyde (PFA) for Figs. 1 and 2 or (2) room temperature for 20 min using 1.6% PFA for Fig. 6 and Extended Data Figs. 5 and 6. Fixed cells were permeabilized and blocked for 1 h using blocking buffer (5% BSA, 0.1% Triton X-100) and incubated overnight at 4 °C with primary antibody: anti-αSMA (1:100, Abcam, ab7817), anti-periostin (1:50, R&D Systems, AF2966), anti-FLAG (1:100, Sigma-Aldrich, B3111), anti-H1.0 (1:100, Abcam, ab134914), anti-vimentin (1:200, Abcam, ab45939) or anti-lamin A/C (1:200, Abcam, ab8984). Appropriate concentration of secondary antibodies was incubated at room temperature for 1 h. Imaging was performed on a fluorescence microscope (Zeiss Axio Vert.A1) or a confocal microscope (Nikon, A1R, ×60). Nuclei were stained using DAPI. Secondary antibody staining alone was used as a negative control.

### Bulk RNA-seq and bioinformatics analysis

Pellets from three biological replicates of primary isolated cardiac fibroblasts transfected with H1.0 or scrambled siRNAs and treated with TGF-β or vehicle were sent to the UCLA Technology Center for Genomics & Bioinformatics Core for RNA isolation, library preparation and sequencing. Ribosomal RNA was removed using a KAPA RNA HyperPrep Kit (Roche, kk8561). Approximately 40 million paired-end reads per sample (2×150 bp) were generated during sequencing. Raw fastq.gz files were downloaded and processed as described^67^, with the following modifications. Salmon version 1.4.0 (ref. ^68^) was used to pseudoalign reads to an mm10 index built from Ensembl build 102. DESeq2 (ref. ^69^) was used to perform differential expression testing, specifically on genes that had at least 10 reads measured between the total samples, and significantly differentially expressed genes were defined as those with adjusted *P* value less than 0.01. Principal component analysis (PCA) was performed using the ‘plotPCA()’ function in DESeq2 and visualized using ggplot2 (ref. ^70^) in R. Heatmaps were visualized using gplots (https://cran.r-project.org/web/packages/gplots/index.html) in R or Prism software version 9.0 (GraphPad Software). KEGG pathway analysis was performed using g:Profiler^71^ on the subset of genes upregulated by log_2_fold change of 1.5 with TGF-β (when compared to scrambled negative control) and then downregulated by log_2_fold change of 1.5 in the H1.0 siRNA + TGF-β condition (when compared to TGF-β alone).

### RT–qPCR

Total RNA was isolated from primary cardiac fibroblasts or primary lung fibroblasts from C57BL/6J mice using an RNA isolation kit (Zymo, R1018) for RT–qPCR. Heart tissue from C57BL/6J mice was homogenized and lysed with TRIzol (Thermo Fisher Scientific, 15596018). Total RNA was extracted following the manufacturer’s instructions. cDNA was generated according to the manufacturer’s instructions (Bio-Rad, 1708891), and RT–qPCR was performed in a CFX96 Real-Time PCR Detection System (Bio-Rad) using SsoFast EvaGreen Supermix (Bio-Rad, 1725201). All primer sequences used in this study are listed in Supplementary Table 2.

### RNAi assay in vitro

Lipofectamine RNAiMAX (Thermo Fisher Scientific, 13778150) transfection was performed following the manufacturer’s protocols. In brief, 500 µl of Opti-MEM Reduced Serum Media (Thermo Fisher Scientific, 31985070) containing either Dharmacon’s siRNA targeting H1.0 (40 nM, Horizon Discovery, M-060325-01), H1.1 (80 nM, Horizon Discovery, M-049956-00), H1.2 (80 nM, Horizon Discovery, M-045246-00), H1.3 (80 nM, Horizon Discovery, M-051171-00), H1.4 (80 nM, Horizon Discovery, M-042536-01), H1.5 (80 nM, Horizon Discovery, M-049995-00) and Thbs4 (40 nM, Horizon Discovery, M-044016-01) or the appropriate concentration of siRNA scramble control (Horizon Discovery, D-001206-14) were mixed with 500 µl of Opti-MEM Reduced Serum Media containing 20 µl of Lipofectamine RNAiMAX (Thermo Fisher Scientific, 13778075). For the human skin fibroblast experiment, transfection was performed using Dharmacon’s siRNA targeting human H1.0 (40 nM, Horizon Discovery, M-017209-01). After incubating the reagents for 10 min at 37 °C, the solution was added to the cells and slightly agitated to mix. After 24 h of incubation at 37 °C, the siRNA reagent solution was removed and replaced with appropriate media according to the downstream experiment. siRNA sequences are listed in Supplementary Table 2.

### Viral infection in isolated cells

Isolated cardiac fibroblasts were infected with either mouse Adv-GFP-H1.0-FLAG (Vector Biolabs, custom generated for this study) or human Adv-HDAC1 (Vector Biolabs, 1498) with Adv-GFP (Vector Biolabs, 1768) as a negative control, using a multiplicity of infection (MOI) of 200 plaque-forming units per cell. After 24 h of incubation at 37 °C, the solution was removed and replaced with DMEM/F12 media containing 10% FBS, 1% antibiotics (penicillin and streptomycin) and 0.1% ITS. For the Adv-GFP-H1.0-FLAG experiments, cells were collected 48 h after infection. In the case of the human Adv-HDAC1 experiments, an additional infection was performed 24 h after initial infection, and cells were collected for downstream analyses 24 h later, for a total of 48 h of infection.

### AAV9 delivery in vivo

AAV9-Tcf21-GFP-shRNAmir encoding shRNA against histone H1.0 was custom generated by Vector Biolabs for this study with sequences (shRNAmir) optimized to facilitate transcription of small non-coding RNAs, including shRNA. A separate vector, encoding a scrambled shRNA, was used as control. Transfection was validated by GFP fluorescence and histone H1.0 abundance. AAV9 (3 × 10^11^ particles per mouse) was injected via tail vein into C3H/HeJ mice (8–12 weeks). Five weeks after injection, PBS or ISO (20 mg kg^−1^ day^−1^) were injected for 1 week. Mice were euthanized 7d after ISO treatment and then hearts were harvested. A similar strategy was employed to target cardiac fibroblasts previously^72^. shRNA sequences are listed in Supplementary Table 2.

### Collagen gel contraction assay

Primary cardiac fibroblasts were transfected with H1.0 or scrambled negative control siRNA for 48 h. Fibroblasts suspended in 10% serum-supplemented DMEM/F-12 medium were seeded (0.5 × 10^6^ cells per milliliter) on collagen gels 24 h before serum deprivation for 4 h. At the beginning of contraction, gels were released from wells using a pipette tip and treated with TGF-β (10 ng ml^−1^; Novoprotein, CA59) for 24 h. Primary cardiac fibroblasts transfected with Adv-GFP or Adv-GFP-H1.0-FLAG for 48 h were suspended in 10% serum-supplemented DMEM/F-12 medium, seeded (0.5 × 10^6^ cells per milliliter) on collagen gels for 8 h and then released from wells for 24 h. Gel images were acquired by the ChemiDoc MP Imaging System (Bio-Rad). Gel area was calculated using ImageJ^73^ and Fiji^74^, and contraction was reported as percentage of contraction.

### Traction force assay

Primary cardiac fibroblasts transfected with Cy3-labeled siRNA (Horizon Discovery, D-001620-03), together with either scrambled or H1.0 siRNA with or without TGF-β stimulation, were seeded onto BSA conjugated to a 647-fluorophore micropatterned onto a flexible polydimethylsiloxane (PDMS). Fibroblasts and patterned BSA dots were imaged, and deformation of the dots was quantified and converted into forces as described^28^.

### CCK-8 proliferation assay

Primary cardiac fibroblasts were seeded in a 48-well plate (1 × 10^4^ cells per well) overnight, transfected with H1.0 or scrambled siRNA for 48 h and then treated with TGF-β (10 ng ml^−1^) or vehicle for 24 h. After 2 h of incubation with 20 µl of CCK-8 solution to each well, absorbance at 450 nm wavelength was recorded in a BioTek Synergy H1 Hybrid plate reader as a readout for cell proliferation.

### Wound healing assay

Wound healing experiments were performed on (1) primary mouse cardiac fibroblasts transfected with Adv-GFP-H1.0-FLAG or Adv-GFP for 48 h; (2) primary murine cardiac fibroblasts transfected with H1.0 or scrambled siRNA for 48 h and then treated with TGF-β (10 ng ml^−1^) or vehicle for 24 h; or (3) human skin fibroblasts from passage 9 transfected with H1.0 or scrambled siRNA for 48 h. After transfection and/or TGF-β treatment, when cells were around 100% confluency, a scratch was made in the culture plate using a P200 pipette tip. Images were taken at 0 h and 24 h using a microscope (Zeiss Axio Vert.A1), and the percentage of wound closure was calculated using ImageJ^73^ and Fiji^74^.

### IPA

Based on differential gene expression analysis of RNA-seq data detailed above, core analysis was applied in IPA to identify potential upstream regulators by comparing gene expression in scramble+TGF-β versus scramble and H1.0 siRNA+TGF-β versus scramble+TGF-β groups. The activation z-score (z ≥ 2 indicates activation or z ≤ −2 indicates inhibition) was applied to predict activation or inhibition state of upstream regulators. The related gene expression changes and pathways with upstream regulators are displayed in Fig. 3 to illustrate a possible mechanistic network in TGF-β-treated cardiac fibroblasts. The same genes and pathways are displayed for the H1.0 siRNA+TGF-β group. The Path Designer tool within IPA was used to visualize these networks. For ease of visualization, some genes appear without lines (Ingenuity Systems).

### Immunohistology

Cardiac tissue samples were fixed in 10% formalin-buffered solution (Sigma-Aldrich, HT501128) overnight, dehydrated in 70% ethanol and sent to the UCLA Translational Pathology Core to generate paraffin blocks. Samples were cut into 4-µm-thick slices, put on slides and stained with hematoxylin and eosin or Masson’s trichrome stain (Sigma-Aldrich, HT15-1KT) to detect fibrosis. Fibrotic area for each slide was quantified and expressed as the percentage of the area occupied by the whole heart on a given slide. For kidney and lung fibrosis quantification, where the whole organ was not able to be imaged at high resolution within the same field of view, five images were taken from each mouse using ×10 magnification (Zeiss Axio Vert.A1). ImageJ^73^ and Fiji^74^ were used to calculate fibrotic area.

### Cardiac fibrosis quantification

For cardiac fibrosis quantification, whole mouse hearts were cut into 2–3 pieces (~2 mm in size). Subsequently, each piece was sectioned into 4-µm-thick slices, mounted onto slides and subjected to staining with hematoxylin and eosin or Masson’s trichrome stain (Sigma-Aldrich, HT15-1KT) to detect fibrosis. The area of fibrosis was determined using color thresholding in ImageJ. The quantitative data presented are from 2–3 histological sections taken from two or more regions of ventricle, for a total of 5–6 images per heart.

### Kidney fibrosis quantification

For kidney fibrosis quantification, where the whole organ was not able to be imaged at high resolution within the same field of view, five images in TIFF format were randomly taken from each mouse using ×10 magnification. ImageJ and Fiji were used to calculate fibrotic area. Specifically, the color threshold option from Fiji was applied to set the appropriate threshold to distinguish Masson’s trichrome staining (deep blue) from background staining as follows, for each image: Fibrotic Area % = Deep Blue Area / Total Area.

### Targeted nuclease digestion and RT–qPCR

Chromatin accessibility in isolated cardiac fibroblasts or whole heart from C57BL/6J mice was measured using the Chromatin Accessibility Assay Kit (Abcam, ab185901) according to the manufacturer’s instructions. After chromatin digestion and DNA purification, a TapeStation 4200 (Agilent) was used to visualize DNA fragment size and intensity. Open chromatin is easily accessed by nucleases and digested more frequently, thereby showing a lower qPCR amplification signal relative to less accessible regions. To assess chromatin accessibility at specific loci within the periostin and αSMA promoters by RT–qPCR, three independent primer sets were designed for each promoter (sequences are provided in Supplementary Table 2). Undigested DNA was used as a negative control.

### Co-IP

Primary murine cardiac fibroblasts were transfected with Adv-GFP-H1.0-FLAG or Adv-GFP for 48 h. The co-IP assay was performed using a FLAG Immunoprecipitation Kit (Sigma-Aldrich, FLAGIPT1-1KT) according to the manufacturer’s instructions. Immunoprecipitated proteins were eluted using the SDS sample buffer included in the kit and then subjected to immunoblotting.

### Hypotonic and hypertonic treatment of cells

Isolated murine cardiac fibroblasts were exposed to (1) a 1:1 mix of DMEM/F12 media supplemented with 10% FBS, 1% antibiotics (penicillin and streptomycin) and 0.1% ITS and water to reach a concentration of 140 mOsm (hypotonic treatment) or (2) a mix of DMEM/F12 media supplemented with 10% FBS, 1% antibiotics (penicillin and streptomycin), 0.1% ITS and 10× PBS (2 ml of media mixed with 213 μl of PBS) to reach a concentration of 560 mOsm (hypertonic treatment). One hour after treatment, cells were fixed using 1.6% PFA solution in PBS.

### Image analysis of CCP

Primary mouse cardiac fibroblasts were fixed with 1.6% PFA and stained with DAPI. Nuclear images were taken using a confocal microscope (Nikon, A1R, ×60). Images were converted to 8-bit format, and each individual nucleus was cropped from the image field by the Fiji package within ImageJ^73,74^. CCP was calculated using a previously published MATLAB script^45,75^. In brief, the Sobel edge detection algorithm was applied to define edges within the nucleus. The density of edges within nucleus was then normalized to its cross-sectional area, giving the measured level of chromatin condensation.

### Cellular deformability assay

To measure the deformability of cardiac fibroblasts under different treatment conditions, suspended cells were filtered by air pressure through a 10-µm porous membrane (Millipore) on timescales of seconds using cellular microfiltration as previously described^44^. Cell deformability was determined by measuring the retention volume after 2–4 min of applied pressure. Large volume retained indicates that cells are less deformable. Small volume retained indicates that cells are more deformable. Before the filtration assay, cell viability (trypan blue staining) and cell size were measured by an automated cell counter (TC20, Bio-Rad). To perform the assay, 400 µl of cell suspension (5 × 10^5^ per milliliter) was loaded into each well of a 96-well plate. The absorbance of retained cell volume was measured at 562 nm by a plate reader (SpectraMax, M2). Retention was determined by the retained volume of cells divided by the initial volume.

### ChIP-seq and bioinformatics analysis

#### H1.0 and FLAG ChIP-seq

Primary isolated murine cardiac fibroblasts were transfected with Adv-GFP-H1.0-FLAG for 48 h. FLAG and H1.0 chromatin immunoprecipitation was performed using anti-FLAG (Sigma-Aldrich, F1804) and anti-H1.0 (Proteintech, 17510-1-AP) antibodies. In a separate experiment, primary isolated mouse cardiac fibroblasts were transfected with H1.0 siRNA or scramble for 72 h. Chromatin shearing was performed using the truChIP Chromatin Shearing Kit (Covaris, 520154) according to the manufacturer’s instructions. DNA fragment size was assessed using a TapeStation 4200 (Agilent). Samples in the 300–500-bp range were used for immunoprecipitation using the ChIP-IT High Sensitivity Kit (Active Motif, 53040). DNA was purified using a Zymo DNA Clean & Concentrator-5 Kit (Zymo, D4014). Library preparation and DNA sequencing were performed by the UCLA Technology Center for Genomics & Bioinformatics Core. Approximately 35 million paired-end reads per sample (2×150 bp) were generated and used for bioinformatic analyses. Alignment of paired-end reads to the mm10 genome was performed as described^67^. After using the ‘bamCoverage’ function of deepTools^76^ with parameters --smoothLength 150 and --normalizeUsing RPGC to generate log_2_(IP/input) bigWig tracks, the ‘computeMatrix’ function was used with parameters -b 5000 -a 5000 --binSize 250 --nanAfterEnd–referencePoint TSS --skipZeros to calculate occupancy around TSSs of genes upregulated, downregulated and unchanged with a given biological treatment. Visualization of occupancy as a ChIP-seq profile was performed using the ‘plotProfile’ function of deepTools version 3.0.2 with default parameters.

#### H3K27ac ChIP-seq

Three biological replicates of isolated cardiac fibroblasts from passage 1 were transfected with H1.0 or scrambled siRNAs for 48 h and treated with TGF-β (10 ng ml^−1^) for 24 h. H3K27ac immunoprecipitation was performed using the same experimental strategy as the H1.0 ChIP-seq experiment but using an anti-H3K27ac antibody (Abcam, ab4729). For each biological condition, a combined input sample of sonicated genomic DNA was obtained from all three biological replicates. Library preparation and paired-end sequencing were performed at the UCLA Technology Center for Genomics and Bioinformatics Core, resulting in approximately 50–70 million read pairs (2×150 bp) per sample. Alignment was performed against the mm10 genome using Bowtie 2 (ref. ^77^) followed by SAM-to-BAM conversion and sorting using SAMtools version 1.7 (ref. ^78^).

Peak calling for each sample was performed using MACS version 2.2.7.1 (ref. ^79^), using the ‘callpeak’ function with the following layout and parameters: --treatment ChIP_replicate.sorted.bam --control Input_sorted.bam -f BAMPE -g mm. Differential occupancy of H3K27ac was determined using the ‘DiffBind’ package^80^ version 3.6.1 in R, specifically on a set of consensus peaks measured in at least three samples across our experiment. We defined significantly differentially occupied regions as those with false discovery rate (FDR) < 0.05. To visualize H3K27ac signal in differentially occupied (FDR < 0.05) regions with TGF-β that undergo an opposite change (no thresholding) in H3K27ac signal with H1.0 depletion, we used a heatmap visualization. For each biological condition, we merged read alignments from all three biological replicates using the SAMtools version 1.7 ‘merge’ function, followed by sorting using the SAMtools version 1.7 (ref. ^78^) ‘sort’ function, and then generated genome browser tracks (bigWig files) of the log_2_fold change in signal using the ‘bamCompare’ function of deepTools version 3.0.2 with the --smoothLength 150, --outFileFormat bigwig -b1 treatment_merged.bam and -b2 control_merged.bam parameters. These bigWigs were used as inputs for the ‘computeMatrix’ function of deepTools^76^ version 3.0.2 with the following parameters: reference-point --referencePoint center -b 5000 -a 5000 --skipZeros. We then used the matrix output of ‘computeMatrix’ as input for the ‘plotHeatmap’ function of deepTools version 3.0.2 with –zMin −1.2 and–zMax 1.2, which generated the final heatmap visualization of the H3K27ac ChIP-seq data. The subset of closest genes to these regions of interest, whose expression increases (no thresholding) with TGF-β (compared to scrambled control) and decreases (no thresholding) in the H1.0 siRNA+TGF-β condition (compared to TGF-β alone), was examined by g:Profiler^71^ using an adjusted *P* value threshold of less than 0.05.

### ChIP–qPCR

ChIP–qPCR against histone H1.0 or BRD4 was performed using the chromatin immunoprecipitation method described above for ChIP-seq but with qPCR as the endpoint. For ChIP–qPCR measurements at specific loci, eluted immunoprecipitated DNA was used to perform qPCR using primer sets designed to amplify specific regions of the periostin, αSMA or Sertad4 promoters. Primer sequences and antibodies are listed in Supplementary Tables 1 and 2. Primers against the GAPDH promoter were used as a positive control (Active Motif, 71018).

### Single Cell RNA-seq bioinformatics analysis

Figure 1a,b was generated from the scRNA-seq data website https://www.fibroxplorer.com/ (ref. ^21^), using 100 random cells per indicated condition. To generate Fig. 1c, data were downloaded from the National Center for Biotechnology Information (NCBI) (GSE120064)^24^. Reads aligning to predicted genes and mitochondrial transcripts (those beginning with ‘Gm’ and ‘mt-’, respectively) were removed from the unique molecular identifier (UMI) matrix. Seurat version 4.0.1 (ref. ^81^) was used to create a Seurat object and perform all downstream analyses. The Seurat object was split by sample and normalized, and the 3,000 most variable features were identified in each dataset with SCTransform^82^. The Seurat objects corresponding to each sample were then integrated together with iterative pairwise integration^83^. PCA was performed for the first 50 principal components of the integrated object. An elbow plot was used to determine the dimensions to use (16) for identifying neighbors and clustering. The *k*-nearest neighbors and shared nearest neighbor graph for the dimensionality-reduced dataset was computed with FindNeighbors, with the default k.param of 20. Louvain clustering was performed with FindClusters, with a resolution of 1.2 and visualized by uniform manifold approximation and projection (UMAP). With default assay set to ‘RNA’, feature counts were normalized by cell (LogNormalize method of NormalizeData), and features were centered and scaled by standard deviation (ScaleData) for downstream differential gene expression analysis. Markers of each cluster were identified with the Wilcoxon rank-sum test via FindAllMarkers, comparing each cluster to all other cells, only testing genes detected in at least 25% of cells in either the cluster of interest or the other cells and only returning genes with *P* < 0.05. Cell type clusters were determined by gene expression levels of markers in Fig. 1e from Ren et al.^24^. H1 isoform (‘H1f0’, ‘Hist1h1a’, ‘Hist1h1c’, ‘Hist1h1d’, ‘Hist1h1e’, ‘Hist1h1b’ and ‘H1fx’) expression levels in each cell type were calculated with AverageExpression.

For Extended Data Fig. 1b,f, data were downloaded from the NCBI (GSE109816 and GSE121893)^23^. UMI and metadata tables from both sources were merged and intersected, respectively. Because there are cells in the UMI matrix for which metadata are not available, the UMI matrix was trimmed to include only those cells for which there are associated metadata. The UMI matrix and metadata were further filtered to include only the following: cells that express more than 500 genes per cell; cells for which UMI count was within 2 s.d. from the mean of log_10_(UMI) of all cells; cells with mitochondrial gene expression (‘MT-’) less than 72%; and cardiomyocytes with sufficient UMIs (over 10,000). Lastly, all mitochondrial genes (‘MT-’) were filtered from the UMI matrix. Seurat version 4.0.1 was used to create a Seurat object of the remaining 10,077 cells and perform all downstream analyses. The Seurat object was split by sample, and each of the 20 samples was normalized with Seurat’s NormalizeData function, in which feature counts for each cell are divided by the total counts for that cell and multiplied by 10,000 before being natural log transformed using log1p. The most variable features in each dataset were identified by dividing features into 20 bins based on average expression and calculating z-scores for dispersion within each bin with FindVariableFeatures (selection.method = ‘mvp’). These variable features were then used for iterative pairwise integration^83^. PCA was performed for the first 50 principal components of the integrated object. The *k*-nearest neighbors and shared nearest neighbor graph for the dimensionality-reduced dataset was computed with FindNeighbors, with 10 dimensions used and the default k.param of 20. Louvain clustering was performed with FindClusters, with a resolution of 1 and visualized by UMAPPlot. To identify cardiac fibroblast cells, the default assay was changed back to ‘RNA’, and feature counts were normalized by cell (LogNormalize method of NormalizeData), and features were centered and scaled by standard deviation (ScaleData) for downstream differential gene expression analysis. For Extended Data Fig. 1b, differential gene expression in each cluster was identified with the Wilcoxon rank-sum test via FindAllMarkers, comparing each cluster to all other cells, only testing genes that are detected in at least 20% of cells in either the cluster of interest or the other cells and only returning genes with *P* < 0.05. Cell type clusters were determined by gene expression levels of markers in ref. ^23^. H1 isoform (‘H1F0’, ‘HIST1H1A’, ‘HIST1H1C’, ‘HIST1H1D’, ‘HIST1H1E’, ‘HIST1H1B’ and ‘H1FX’) expression levels in each cell type were calculated with AverageExpression. For Extended Data Fig. 1f, the dataset was further subset to just those fibroblasts with non-zero expression of POSTN and H1F0, and the expression of POSTN and H1F0 in the resulting 51 cells was reported with FetchData. Scatter plots were plotted with ggpubr, and Spearmanʼs correlation was calculated with ‘stat_cor()’.

For human data in Extended Data Fig. 1d, snRNA-seq data were downloaded from the Chan Zuckerberg CELLxGENE Discover database (https://cellxgene.cziscience.com/collections/8191c283-0816-424b-9b61-c3e1d6258a77). To convert the uploaded .h5ad file to a Seurat object, Scanpy was used to run ‘sc.read_h5ad()’ and create a folder of matrix, features and barcode files for import into Seurat version 4.0.1. Cardiac fibroblast nuclei, as identified by the associated metadata, were subset from the object. With default assay set to ‘RNA’, feature counts were normalized by nucleus (LogNormalize method of NormalizeData), and features were centered and scaled by standard deviation (ScaleData) for downstream differential gene expression analysis. The dataset was further subset for those fibroblasts with non-zero expression of POSTN and H1F0, and the expression of POSTN and H1F0 in the resulting 345 nuclei was reported with FetchData. Scatter plots were plotted with ggpubr, and Spearmanʼs correlation was calculated with ‘stat_cor()’.

For human data in Extended Data Fig. 1e, the scRNA-seq data were downloaded as a Seurat object from the NCBI (GSE183852). In total, 23,549 cardiac fibroblast cells, as identified by the associated metadata, were subset from the object. With default assay set to ‘RNA’, feature counts were normalized by cell (LogNormalize method of NormalizeData), and features were centered and scaled by standard deviation (ScaleData) for downstream differential gene expression analysis. The dataset was further subset for those fibroblasts with non-zero expression of POSTN and H1F0, and the expression of POSTN and H1F0 in the resulting 1,257 cells was reported with FetchData. Scatter plots were plotted with ggpubr, and Spearmanʼs correlation was calculated with ‘stat_cor()’.

### Quantification and statistical analysis

Data are presented as the mean ± s.d., unless otherwise indicated in the figure legends. Statistical analyses were performed using Prism software version 9.0 (GraphPad Software) using Welch’s *t*-test between two groups and one-way ANOVA with Tukey’s multiple comparison analysis among three or more groups. *P* values less than 0.05 were considered statistically significant. Two-sided tests were performed, and all ‘replicates’ are biological replicates, meaning from different animals or cell isolations (depending on the type of experiment), unless otherwise noted.

### Reporting summary

Further information on research design is available in the Nature Portfolio Reporting Summary linked to this article.

### Supplementary information

Reporting Summary

Supplementary Table 1 List of reagents, antibodies and biochemical tools used in this study.

Supplementary Table 2 List of primers and siRNA used in this study.

### Source data

Source Data Fig. 1 Measurements for graphs in Fig. 1.

Source Data Fig. 1 Unprocessed images and blots in Fig. 1.

Source Data Fig. 2 Measurements for graphs in Fig. 2.

Source Data Fig. 2 Unprocessed images and blots in Fig. 2.

Source Data Fig. 3 Measurements for graphs in Fig. 3.

Source Data Fig. 3 Unprocessed images and blots in Fig. 3.

Source Data Fig. 4 Measurements for graphs in Fig. 4.

Source Data Fig. 4 Unprocessed images and blots in Fig. 4.

Source Data Fig. 5 Measurements for graphs in Fig. 5.

Source Data Fig. 6 Measurements for graphs in Fig. 6.

Source Data Fig. 7 Measurements for graphs in Fig. 7.

Source Data Fig. 7 Unprocessed images and blots in Fig. 7.

Source Data Extended Data Fig. 2 Measurements for graphs in Extended Data Fig. 2.

Source Data Extended Data Fig. 2 Unprocessed images and blots in Extended Data Fig. 2.

Source Data Extended Data Fig. 3 Measurements for graphs in Extended Data Fig. 3.

Source Data Extended Data Fig. 3 Unprocessed images and blots in Extended Data Fig. 3.

Source Data Extended Data Fig. 4 Measurements for graphs in Extended Data Fig. 4.

Source Data Extended Data Fig. 4 Unprocessed images and blots in Extended Data Fig. 4.

Source Data Extended Data Fig. 5 Measurements for graphs in Extended Data Fig. 5.

Source Data Extended Data Fig. 5 Unprocessed images and blots in Extended Data Fig. 5.

Source Data Extended Data Fig. 6 Measurements for graphs in Extended Data Fig. 6.

Source Data Extended Data Fig. 7 Measurements for graphs in Extended Data Fig. 7.

Source Data Extended Data Fig. 7 Unprocessed images and blots in Extended Data Fig. 7.

Source Data Extended Data Fig. 8 Measurements for graphs in Extended Data Fig. 8.

Source Data Extended Data Fig. 9 Measurements for graphs in Extended Data Fig. 9.

Source Data Extended Data Fig. 9 Unprocessed images and blots in Extended Data Fig. 9.

## Data Availability

Raw and processed RNA-seq and ChIP-seq data generated during this study were deposited in the National Center for Biotechnology Informationʼs Gene Expression Omnibus and are available for download using accession number GSE215268. Source data are provided with this paper.

## Code availability

All software used in this study was from public software packages described in Methods and detailed in the accompanying references.
