# Supplementary material for: Histone H1.0 couples cellular mechanical behaviors to chromatin structure
Source: Nat Cardiovasc Res. 2024 Apr 10;3(4):441–59. doi: 10.1038/s44161-024-00460-w (PMC11101354; doi:10.1038/s44161-024-00460-w)
Supplement: Supplementary file 1 — Reporting Summary [file 44161_2024_460_MOESM1_ESM.pdf]

nature portfolio

Corresponding author(s): Thomas Vondriska

Last updated by author(s): 2024/03/03

Reporting Summary

Nature Portfolio wishes to improve the reproducibility of the work that we publish. This form provides structure for consistency and transparency in reporting. For further information on Nature Portfolio policies, see our Editorial Policies and the Editorial Policy Checklist.

Statistics

For all statistical analyses, confirm that the following items are present in the figure legend, table legend, main text, or Methods section.

n/a	Confirmed	
☐	☒	The exact sample size (*n*) for each experimental group/condition, given as a discrete number and unit of measurement
☐	☒	A statement on whether measurements were taken from distinct samples or whether the same sample was measured repeatedly
☐	☒	The statistical test(s) used AND whether they are one- or two-sided<br>*Only common tests should be described solely by name; describe more complex techniques in the Methods section.*
☐	☒	A description of all covariates tested
☐	☒	A description of any assumptions or corrections, such as tests of normality and adjustment for multiple comparisons
☐	☒	A full description of the statistical parameters including central tendency (e.g. means) or other basic estimates (e.g. regression coefficient) AND variation (e.g. standard deviation) or associated estimates of uncertainty (e.g. confidence intervals)
☐	☒	For null hypothesis testing, the test statistic (e.g. *F*, *t*, *r*) with confidence intervals, effect sizes, degrees of freedom and *P* value noted<br>*Give P values as exact values whenever suitable.*
☒	☐	For Bayesian analysis, information on the choice of priors and Markov chain Monte Carlo settings
☒	☐	For hierarchical and complex designs, identification of the appropriate level for tests and full reporting of outcomes
☐	☒	Estimates of effect sizes (e.g. Cohen's *d*, Pearson's *r*), indicating how they were calculated

Our web collection on statistics for biologists contains articles on many of the points above.

Software and code

Policy information about availability of computer code

Data collection	All data collection tools and methods are publicly available and described in detail, with references, in the methods section of the paper.
Data analysis	All data analysis tools and methods are publicly available and described in detail, with references, in the methods section of the paper.

For manuscripts utilizing custom algorithms or software that are central to the research but not yet described in published literature, software must be made available to editors and reviewers. We strongly encourage code deposition in a community repository (e.g. GitHub). See the Nature Portfolio guidelines for submitting code & software for further information.

Data

Policy information about availability of data

All manuscripts must include a data availability statement. This statement should provide the following information, where applicable:

- Accession codes, unique identifiers, or web links for publicly available datasets
- A description of any restrictions on data availability
- For clinical datasets or third party data, please ensure that the statement adheres to our policy

Raw and processed RNA-seq and ChIP-seq data generated during this study were deposited in the National Center for Biotechnology Information Gene Expression Omnibus and are available for download using accession number: GSE215268. This dataset was made public Feb 28, 2024.

Research involving human participants, their data, or biological material

Policy information about studies with human participants or human data. See also policy information about sex, gender (identity/presentation), and sexual orientation and race, ethnicity and racism.

Reporting on sex and gender	n/a
Reporting on race, ethnicity, or other socially relevant groupings	n/a
Population characteristics	n/a
Recruitment	n/a
Ethics oversight	n/a

Note that full information on the approval of the study protocol must also be provided in the manuscript.

Field-specific reporting

Please select the one below that is the best fit for your research. If you are not sure, read the appropriate sections before making your selection.

☒ Life sciences ☐ Behavioural & social sciences ☐ Ecological, evolutionary & environmental sciences

For a reference copy of the document with all sections, see nature.com/documents/nr-reporting-summary-flat.pdf

Life sciences study design

All studies must disclose on these points even when the disclosure is negative.

Sample size	Sample sizes were determined based on previous investigations from our lab and others with the respective techniques (including PMID 34273410, 28802249).
Data exclusions	No data were excluded.
Replication	All experiments were reproduced across multiple biological replicates (at least 3 independent experiments).
Randomization	Cells and animals were randomly assigned to experimental or control groups.
Blinding	Aspects of the in vivo experiments were blinded, including histology and echocardiography. Blinding was not used for in vitro experiments as it was not relevant to experimental outcomes.

Reporting for specific materials, systems and methods

We require information from authors about some types of materials, experimental systems and methods used in many studies. Here, indicate whether each material, system or method listed is relevant to your study. If you are not sure if a list item applies to your research, read the appropriate section before selecting a response.

Materials & experimental systems

n/a	Involved in the study	
☐	☒	Antibodies
☐	☒	Eukaryotic cell lines
☒	☐	Palaeontology and archaeology
☐	☒	Animals and other organisms
☒	☐	Clinical data
☒	☐	Dual use research of concern
☒	☐	Plants

Methods

n/a	Involved in the study	
☐	☒	ChIP-seq
☒	☐	Flow cytometry
☒	☐	MRI-based neuroimaging

Antibodies

Antibodies used	All antibody information is provided in the methods section of the manuscript and in Supplementary Table 1.

Validation	All antibody information is provided in the methods section of the manuscript (details regarding the source manufacturer and catalog number are provided and manufacturer validation information is available on their websites) and in Supplementary Table 1.

Eukaryotic cell lines

Policy information about cell lines and Sex and Gender in Research

Cell line source(s)	All cell line information is provided in the methods section of the manuscript.
Authentication	All cell line information is provided in the methods section of the manuscript.
Mycoplasma contamination	All cell line information is provided in the methods section of the manuscript.
Commonly misidentified lines (See ICLAC register)	All cell line information is provided in the methods section of the manuscript.

Animals and other research organisms

Policy information about studies involving animals; ARRIVE guidelines recommended for reporting animal research, and Sex and Gender in Research

Laboratory animals	All mouse information is provided in the methods section of the manuscript. All animal studies were approved by the UCLA Animal Research Committee in compliance with the National Institutes of Health Guide for the Care and Use of Laboratory Animals. Adult female and male C57BL/6J (The Jackson Laboratory, cat. no. 000664) and C3H/HeJ (The Jackson Laboratory, cat. no. 000659) mice (8-12 weeks old) were obtained from Jackson Laboratory and used in the study. Male and female mice were used in this study but the groups were not powered to reveal sex differences.
Wild animals	No wild animals were used for this study.
Reporting on sex	Male and female mice were used in this study but the groups were not powered to reveal sex differences.
Field-collected samples	No field collected samples for this study.
Ethics oversight	All animal studies were approved by the UCLA Animal Research Committee in compliance with the National Institutes of Health Guide for the Care and Use of Laboratory Animals.

Note that full information on the approval of the study protocol must also be provided in the manuscript.

Plants

Seed stocks	n/a
Novel plant genotypes	n/a
Authentication	n/a

ChIP-seq

Data deposition

☒ Confirm that both raw and final processed data have been deposited in a public database such as GEO.

☒ Confirm that you have deposited or provided access to graph files (e.g. BED files) for the called peaks.

Data access links *May remain private before publication.*	GSE215268
Files in database submission	The files are listed in the methods section of the manuscript and at the Gene Expression Omnibus.
Genome browser session (e.g. UCSC)	n/a

Methodology

Replicates	All experiments were performed with at least 3 biological replicates. Exact details are provided in figure legends and methods section of the manuscript.
Sequencing depth	This information is provided in figure legends and methods section of the manuscript.

Antibodies	The use of antibodies is described in the methods and provided here in the attached table.
Peak calling parameters	This information is provided in figure legends and methods section of the manuscript. Data quality was assessed and the mappability percentages are shown in Extended Data Figure 5b.Peak calling was performed with MACS v2.2.7.1, using the callpeak function with the following layout and parameters: --treatment ChIP. replicate.sorted.bam --control Input sorted.bam -f BAMPE -g mm
Data quality	This information is provided in figure legends, supplemental figures and methods section of the manuscript. Data quality was assessed and the mappability percentages are shown in Extended Data Figure 5b.
Software	All software and their utilization are extensively described in the Methods section.
