# Supplementary material for: Histone H1.0 couples cellular mechanical behaviors to chromatin structure
Source: Nat Cardiovasc Res. 2024 Apr 10;3(4):441–59. doi: 10.1038/s44161-024-00460-w (PMC11101354; doi:10.1038/s44161-024-00460-w)
Supplement: Supplementary file 2 — List of reagents, antibodies and biochemical tools used in this study. [file 44161_2024_460_MOESM2_ESM.pdf]

Supplementary Table 1

REAGENT or RESOURCE	SOURCE	Catalog number
**Antibodies**		
Anti-alpha smooth muscle Actin (western blot:1:1000)	Abcam	Cat# ab7817
Mouse Periostin/OSF-2 Isoform 2 (western blot: 1:1000)	R&D Systems	Cat# AF2955
Recombinant Anti-Histone H1.0 (western blot:1.1000)	Abcam	Cat# ab134914
Anti-Histone H1.1 (western blot:1:1000)	Millipore Sigma	Cat#GW20081F
Anti-Histone H1.2 (western blot:1:1000)	Novus	Cat#NBP2-75932
Anti-Histone H1.3 (western blot:1:1000)	Affbiotech	Cat#AF0082
Anti-Histone H1.4 (western blot:1:1000)	Bioss	Cat#bs-10334R
Anti-Histone H1.5 (western blot:1:1000)	LSBio	Cat#LS-B16990
Anti-Glyceraldehyde-3-Phosphate Dehydrogenase (western blot:1:1000)	Millipore Sigma	Cat# MAB374
α-Tubulin (western blot:1:1000)	Cell Signaling	Cat# 2144
GAPDH (V-18) (western blot:1:1000)	Santa Cruz Biotechnology	Cat# sc-20357
Histone H3 (western blot:1:1000)	Cell Signaling	Cat# 9715
Recombinant Anti-THBS4 (western blot:1:1000)	Abcam	Cat# ab263898
RNA pol II CTD phospho Ser2 antibody (western blot:1:1000)	Active motif	Cat# 61083
Anti-Histone H3(acetyl K27) (western blot:1:1000)	Abcam	Cat# ab4729
ChIPAb+ HDAC1 antibody (western blot:1:1000)	Millipore Sigma	Cat# 17-608
Anti-HDAC1 (western blot:1:1000)	Abcam	Cat# ab7028
Monoclonal ANTI-FLAG® M2 (western blot:1:1000)	Millipore Sigma	Cat# F1804
ANTI-FLAG® M2 (western blot:1:1000)	Millipore Sigma	Cat# B3111
Anti-Histone H2A (western blot:1:1000)	Abcam	Cat# ab18255
Anti-Histone H2B (western blot:1:1000)	Abcam	Cat# ab1790
Anti-Histone H4 (western blot:1:1000)	Abcam	Cat# ab10158
DAPI (4',6-Diamidino-2-Phenylindole, Dihydrochloride)	Thermo Fisher Scientific	Cat# D1306
Akt (pan) (C67E7) (western blot:1:1000)	Cell Signaling	Cat# ab4691
Phospho-Akt (Ser473) (D9E) XP® Rabbit mAb (western blot:1:1000)	Cell Signaling	Cat# 4060
RNA pol II (western blot:1:1000)	Active motif	Cat# 39097
Anti-Vimentin antibody - Cytoskeleton Marker	Abcam	Cat# ab45939
Anti-Lamin A + Lamin C antibody [131C3] - Nuclear Envelope Marker	Abcam	Cat# ab8984
Anti-Lamin B1 antibody (western blot:1:1000)	Abcam	Cat# ab16048
Histone H1.0 (western blot:1:1000)	Proteintech	Cat# 17510-1-AP
Anti-BRD4 antibody (western blot:1:1000)	Thermo Fisher Scientific	Cat# A301-985A100
Goat Anti-Rabbit IgG (H + L)-HRP Conjugate (western blot:1:1000)	Bio-Rad	Cat#1706515
IRDye®800CW Goat anti-Mouse IgG Secondary Antibody (western blot:1:2000)	LI-COR	Cat# 926-32210

IRDye®800CW Goat anti-Rabbit IgG Secondary Antibody (western blot:1:2000)	LI-COR	Cat# 926-32211
IRDye®800CW Goat anti-Rat IgG Secondary Antibody (western blot:1:2000)	LI-COR	Cat# 926-32219
IRDye®800CW Donkey anti-Goat IgG Secondary Antibody (western blot:1:2000)	LI-COR	Cat# 926-32214
Goat anti-Rabbit IgG (H+L) Cross-Adsorbed Secondary Antibody, Alexa Fluor™ 488 (immunostaining 1:500)	Thermo Fisher Scientific	Cat# A-11008
Goat anti-Mouse IgG (H+L) Cross-Adsorbed Secondary Antibody, Alexa Fluor™ 647 (immunostaining 1:500)	Thermo Fisher Scientific	Cat# A-21235
Goat anti-Mouse IgG (H+L) Cross-Adsorbed Secondary Antibody, Alexa Fluor™ 568 (immunostaining 1:500)	Thermo Fisher Scientific	Cat# A-11004
Goat anti-Rabbit IgG (H+L) Cross-Adsorbed Secondary Antibody, Alexa Fluor™ 568 (immunostaining 1:500)	Thermo Fisher Scientific	Cat# A-11011
Goat anti-Mouse IgG (H+L) Highly Cross-Adsorbed Secondary Antibody, Alexa Fluor™ 488 (immunostaining 1:500)	Thermo Fisher Scientific	Cat# A-11029
Goat anti-Rabbit IgG (H+L) Cross-Adsorbed Secondary Antibody, Alexa Fluor™ 647 (immunostaining 1:500)	Thermo Fisher Scientific	Cat# A-21244
Recombinant Rabbit IgG, monoclonal [EPR25A] - Isotype Control (immunostaining 1:500)	Abcam	Cat# ab172730
**Chemicals and Recombinant protein**		
Recombinant Mouse Thrombospondin-4	R&D Systems	Cat# 7860-TH
Invivofectamine™ 3.0 Reagent	Thermo Fisher Scientific	Cat# IVF3005
Isoproterenol hydrochloride	Millipore Sigma	Cat# 16504
Collagenase type II	Worthington Biochemical Corporation	Cat# LS004177
Collagenase from Clostridium histolyticum	Millipore Sigma	Cat# C7657
Recombinant Human Transforming Growth Factor β-1/TGFB1	Novoprotein	Cat# CA59
cOmplete™, Mini, EDTA-free Protease Inhibitor Cocktail	Millipore Sigma	Cat# 04693159001
PhosSTOP™	Millipore Sigma	Cat# 04906837001
Pierce™ 16% Formaldehyde (w/v), Methanol-free	Thermo Fisher Scientific	Cat# 28906
TRIzol™ Reagent	Thermo Fisher Scientific	Cat# 15596018
Lipofectamine™ RNAiMAX Transfection Reagent	Thermo Fisher Scientific	Cat# 13778075
Corning™ ITS Premix Universal Culture Supplement	Corning	Cat# 354351
PureCol™ EZ Gel solution	Advanced BioMatrix	Cat# 5074
Angiotension II	Millipore Sigma	Cat# A9525
Gelatin solution	Millipore Sigma	Cat# G1393
Basic Fibroblast Growth Factor,human (hbFGF)	Millipore Sigma	Cat# 11123149001
Formalin solution, neutral buffered, 10%	Millipore Sigma	Cat# HT501128
High Sensitivity D1000 ScreenTape	Agilent Technologies	Cat# 50675584
High Sensitivity D1000 Sample Buffer	Agilent Technologies	Cat# 50675603

Dulbecco's Modified Eagle's Medium/Nutrient Mixture F-12 Ham	Millipore Sigma	Cat# D8437
Glycine	MP Biomedicals	Cat# 808831
Tris Base, Molecular Biology Grade	Calbiochem	Cat# CAS 77-86-1
Opti-MEM™ I Reduced Serum Medium	Thermo Fisher Scientific	Cat# 31985062
ProtoGel (30%)	National Diagnostics	Cat# EC-890
NP-40 Surfact-Amps™ Detergent Solution	Thermo Fisher Scientific	Cat# 28324
Bovine Serum Albumin	Millipore Sigma	Cat# A9647
Fetal Bovine Serum - Premium, Heat Inactivated	Biotechne	Cat# S11150H
Gibco™ Penicillin-Streptomycin (10,000 U/mL)	Gibco Invitrogen	Cat# 15140122
SuperSignal™ West Femto Maximum Sensitivity Substrate	Thermo Fisher Scientific	Cat# 34095
**Critical commercial assays**		
iScript™ cDNA Synthesis Kit	Bio-Rad	Cat# 1708891
SsoFast™ EvaGreen® Supermix	Bio-Rad	Cat# 1725201
Trichrome Stain (Masson) Kit	Millipore Sigma	Cat# HT15—1KT
Cell Counting Kit 8 (WST-8 / CCK8)	Abcam	Cat# 228554
Pierce™ BCA Protein Assay Kit	Thermo Fisher Scientific	Cat# 23225
FLAG® Immunoprecipitation Kit	Millipore Sigma	Cat# FLAGIPT1-1KT
Chromatin Accessibility Assay Kit	Abcam	Cat# ab185901
ChIP-IT High Sensitivity®	Active motif	Cat# 53040
truChIP Chromatin Shearing Kit with Formaldehyde	Covaris	Cat# 520154
RNA Clean & Concentrator-25	Zymo Research	Cat# R1018
DNA Clean & Concentrator-5 kit	Zymo Research	Cat# D4014
