# Supplementary material for: Histone H1.0 couples cellular mechanical behaviors to chromatin structure
Source: Nat Cardiovasc Res. 2024 Apr 10;3(4):441–59. doi: 10.1038/s44161-024-00460-w (PMC11101354; doi:10.1038/s44161-024-00460-w)
Supplement: Supplementary file 3 — List of primers and siRNA used in this study. [file 44161_2024_460_MOESM3_ESM.pdf]

Supplementary Table 2

**RT-qPCR Primers**		
H1f0 (H1.0)	Thermo Fisher Scientific	F: AAGAAGTCCACGGACCACCC<br>R: TGATCTGGGAGTCGGCGTTC
Hist1h1a (H1.1)	Thermo Fisher Scientific	F: GCTGCTTCTACTGCCACGGA<br>R: CGATGAGCTCCGACACCGAA
Hist1h1b (H1.5)	Thermo Fisher Scientific	F: GCCCAAGGCGGTGAAGTCTA<br>R: AGCCTTTGGGGCTTTGTTGC
Hist1h1c (H1.2)	Thermo Fisher Scientific	F: GCCCAAGAAGGTCAAGAGCG<br>R: GTTAGGGGAGGCAGCCTACTT
Hist1h1d (H1.3)	Thermo Fisher Scientific	F: AAGCTTCTAAGCCGAAGGCCA<br>R: AAGCAGGACGCACCACTCT
Hist1h1e (H1.4)	Thermo Fisher Scientific	F: TCTCTCCTCACACGCTTCGC<br>R: GGGCCTTCTTCTTGACGGGT
COL1A1	Thermo Fisher Scientific	F: GCTTGAAGACCTATGTGGGTATAA<br>R: GGTGGAGAAAGGAGCAGAAA
GAPDH	Thermo Fisher Scientific	F: TGTGCAGTGCCAGCCTCGTC<br>R: TGAAGGGGTCGTTGATGGCAACA
**ChIP-qPCR Primers**		
Acta2	Thermo Fisher Scientific	F: GAGGCCTGGGTCTCTTCCA<br>R: GCTGAGCTGCCTCCTGTTTC
Postn	Thermo Fisher Scientific	F: CCACAGCCCAGAGCTATATAAAC<br>R: CAGCAGCAGCAGAGCATATAA
Sertad4	Thermo Fisher Scientific	F: ACAAGACGTCTGGGCAGGT<br>R: AGCCTGGCTCTGCTGGAT
**Nuclear digestion promoter sequence**		
Acta2 (Primer set 1)	Thermo Fisher Scientific	F: ATGAGCCCACTGGACAGAAA<br>R: GCACAGTTCAGAAGCACTCC
Acta2 (Primer set 2)	Thermo Fisher Scientific	F: ACAACTGCTCAAATGCCCAG<br>R: TGGGTGACTCGAAGCATCTT
Acta2 (Primer set 3)	Thermo Fisher Scientific	F: GAGGCCTGGGTCTCTTCCA<br>R: GCTGAGCTGCCTCCTGTTTC
Postn (Primer set 1)	Thermo Fisher Scientific	F: GGAAGAGACTGCTAATTCCTAC<br>R: GAGACATCTAGTGGAGAAAGTG
Postn (Primer set 2)	Thermo Fisher Scientific	F: CCACAGCCCAGAGCTATATAAAC<br>R: CAGCAGCAGCAGAGCATATAA
Postn (Primer set 3)	Thermo Fisher Scientific	F: GGCCTTTACCCTCAGTCACT<br>R: TCCTGAGCCATGTTGACCAT
**siRNA sequences**		
H1f0 (H1.0) (in cells)	Horizon	GGUCGGUGGCUUUCAAGAA,<br>AGAUCAAGUUGUCCAUCAA,<br>CCGUGAAGCCCAAAGCCAA,<br>GUGCCAAGAGGGCCAGCAA
Hist1h1a (H1.1)	Horizon	CCAAGAAGCCUGCAGUUUC,<br>GGCAGUUUCUUCUUCCAAA,<br>ACAAGAAGGCUGAGUCCAA,<br>GCGAAAGUGACAAAGCCAA,
Hist1h1b (H1.5)	Horizon	UCUGGUUCCUUCAAGCUUA,<br>GGGCAAAGAAGACCGUGAA,<br>CCUAAGACCGCUAAGCCUA,<br>GCCAAGAAGAAGACAACGA

Hist1h1c (H1.2)	Horizon	GGAGAAGGCGCCUGCCAAA, GAGCGCGUCUAAAGCCGUA, CGGCUGUGACCAAGAAAGU, GCGAAGGUCACCAAGCCCA
Hist1h1d (H1.3)	Horizon	AGAAGACACCUGUGAAGAA, GCAAAGAGUCCAGCCAAGG, AAGAGUCCCAAGAAGGUGA, GCUCCCAAGGCUAAGGCUU
Hist1h1e (H1.4)	Horizon	GCAAAGGCAACUAAGGCUA, CGAAAACGGUAAAGCCUAA, AAGCUAAGAGCCCGAAGAA, GCAGCUAAGCCAAAGAAAA
H1f0 (H1.0) (in vivo)	Ambion	siRNA, Sense: AGCCCAAGGUUGUCAAAGUtt; Antisense: ACUUUGACAACCUUGGGCUtt
Thbs4	Horizon	CCAGACAGAUGAUGACUAU, GGAUGGUCCUGAAUAAUGA, CGACAGUUCUUGGGUCAAA, GUAGUCAUGUGGAAGCAAA
Scramble	Horizon	No sequence information provided; Cat no. D-001206-14
**AAV9 driven shRNAs**		
H1f0 (H1.0)	Vector Biolabs	GCTGCCACGATCATGTCTGAATACTGTTTTGGCCACTGACTGACAGTATTCACATGATCGTGGCAG
Scramble	Vector Biolabs	CCGGCAACAAGATGAAGAGCACCAACTCGAGTTGGTGCTCTTCATCTTGTTGTTTTT
