# Supplementary material for: Histone H1.0 couples cellular mechanical behaviors to chromatin structure
Source: Nat Cardiovasc Res. 2024 Apr 10;3(4):441–59. doi: 10.1038/s44161-024-00460-w (PMC11101354; doi:10.1038/s44161-024-00460-w)
Supplement: Supplementary file 5 — Unprocessed images and blots in Fig. 1. [file 44161_2024_460_MOESM5_ESM.pdf]

Figure 1d

Control DAPI/periostin

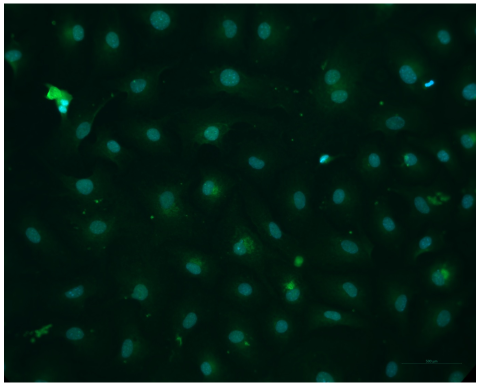

TGF-β teatment DAPI/periostin

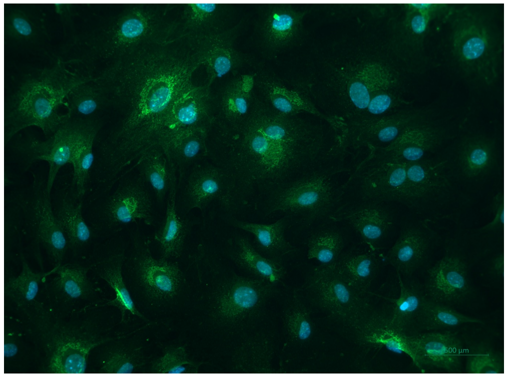

Control DAPI/αSMA

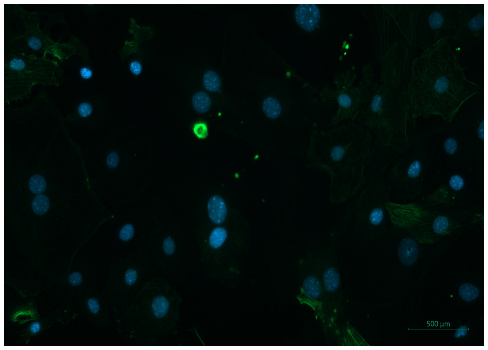

TGF-β teatment DAPI/αSMA

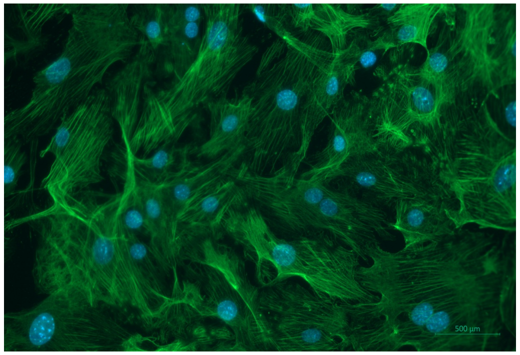

Source Data for: Hu et al. Histone H1.0 Couples Cellular Mechanical Behaviors to Chromatin Structure

Figure 1d

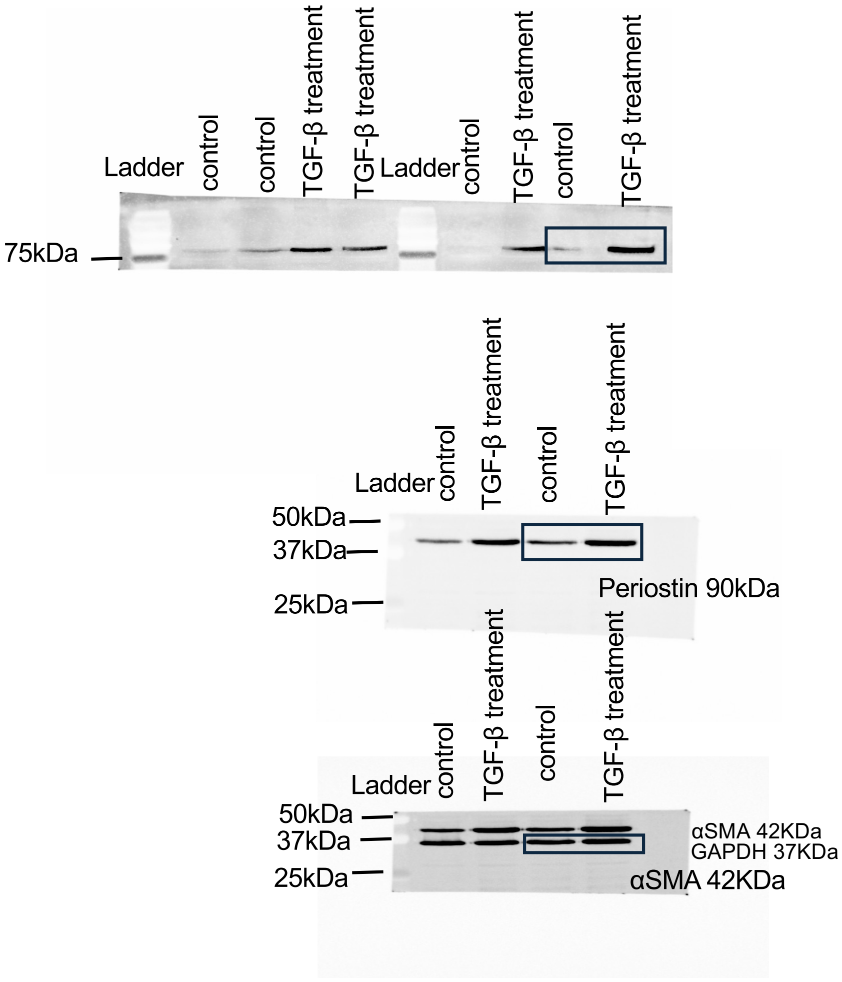

Figure 1e

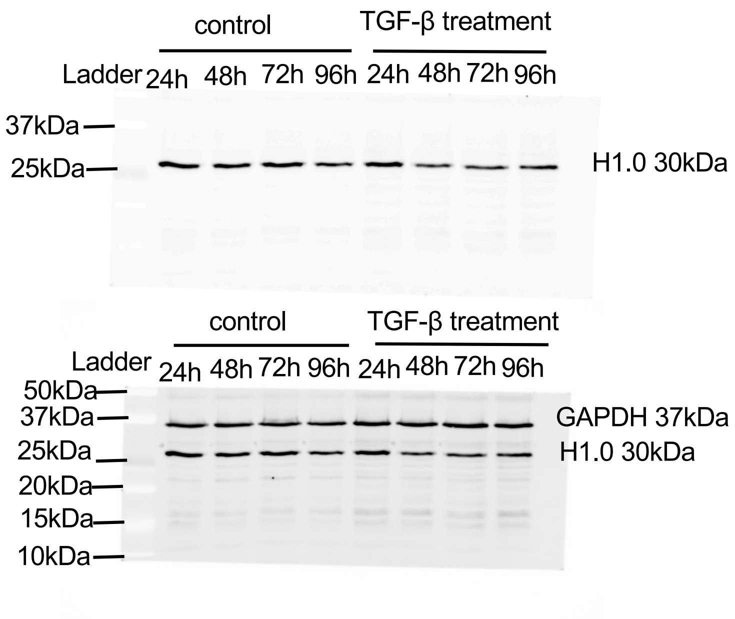

Upper band:αSMA-42kDa
Bottom band: GAPDH-37KDa
After the first day, the membrane was exposed to test αSMA, Subsequently, the same membrane was incubated overnight with the GAPDH primary antibody to measure GAPDH band the next day.

The bands from the blue frame were used for the paper

Source Data for: Hu et al. Histone H1.0 Couples Cellular Mechanical Behaviors to Chromatin Structure
