# Supplementary material for: Histone H1.0 couples cellular mechanical behaviors to chromatin structure
Source: Nat Cardiovasc Res. 2024 Apr 10;3(4):441–59. doi: 10.1038/s44161-024-00460-w (PMC11101354; doi:10.1038/s44161-024-00460-w)
Supplement: Supplementary file 11 — Unprocessed images and blots in Fig. 4. [file 44161_2024_460_MOESM11_ESM.pdf]

Figure 4a

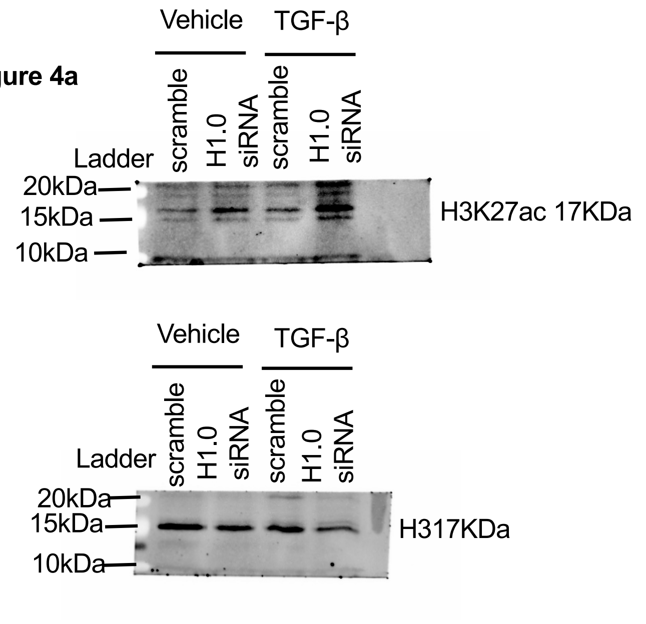

H3K27ac band location is very close, so the same samples were run the same blot but different lanes.

Figure 4d

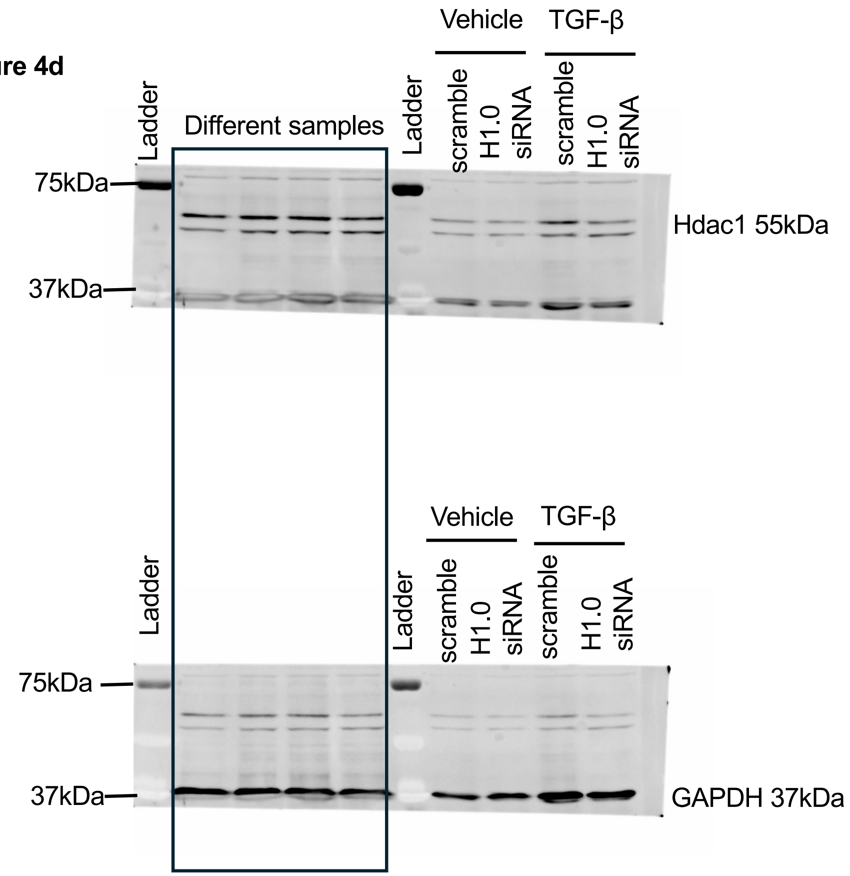

Source Data for: Hu et al. Histone H1.0 Couples Cellular Mechanical Behaviors to Chromatin Structure

Figure 4e

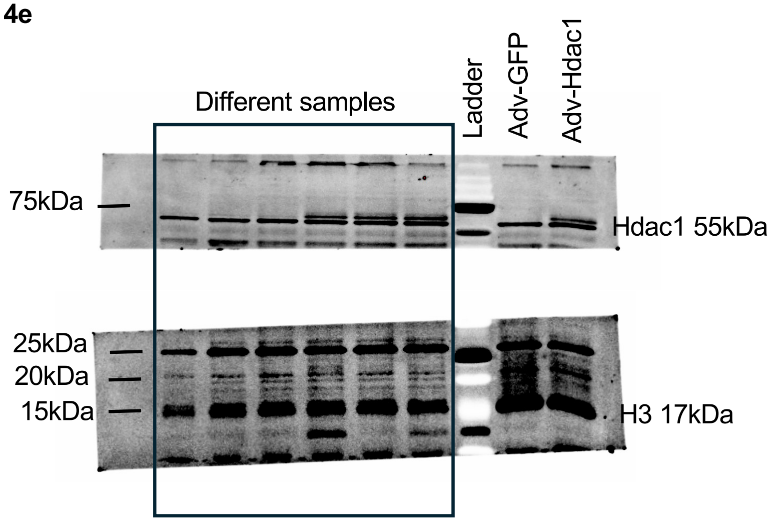

Figure 4f

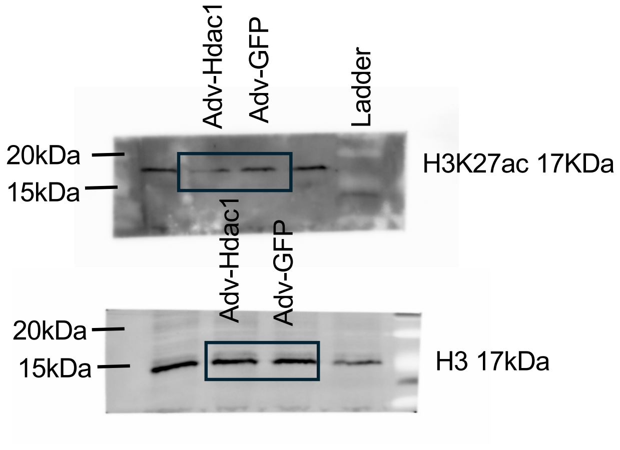

The bands from the blue frame were used for the paper

H3K27ac band location is very close, so the same samples were run the same blot but different lanes.

Figure 4g

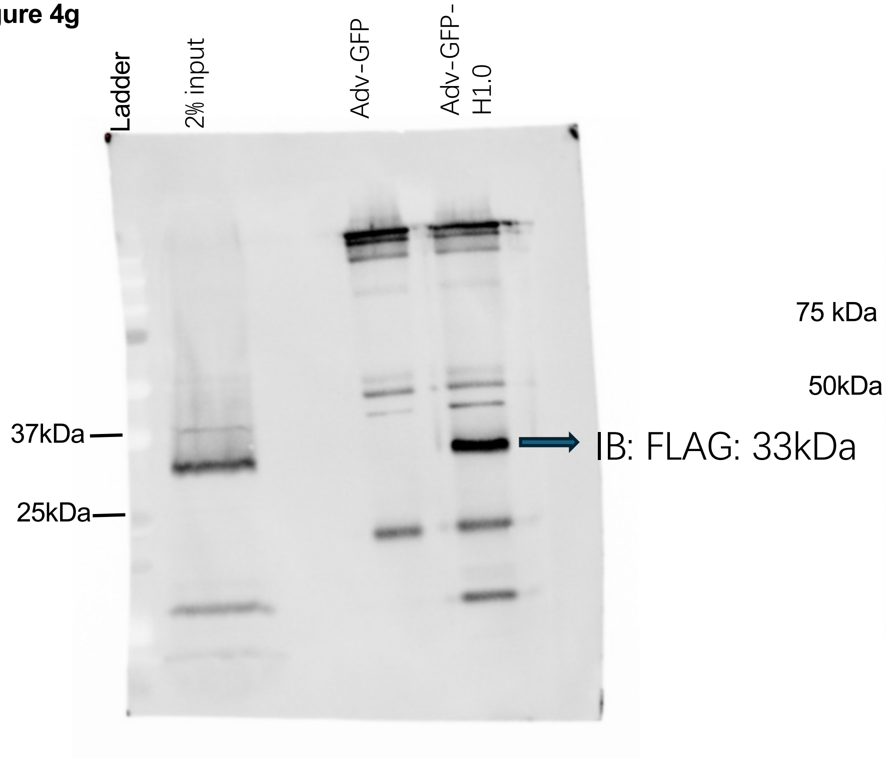

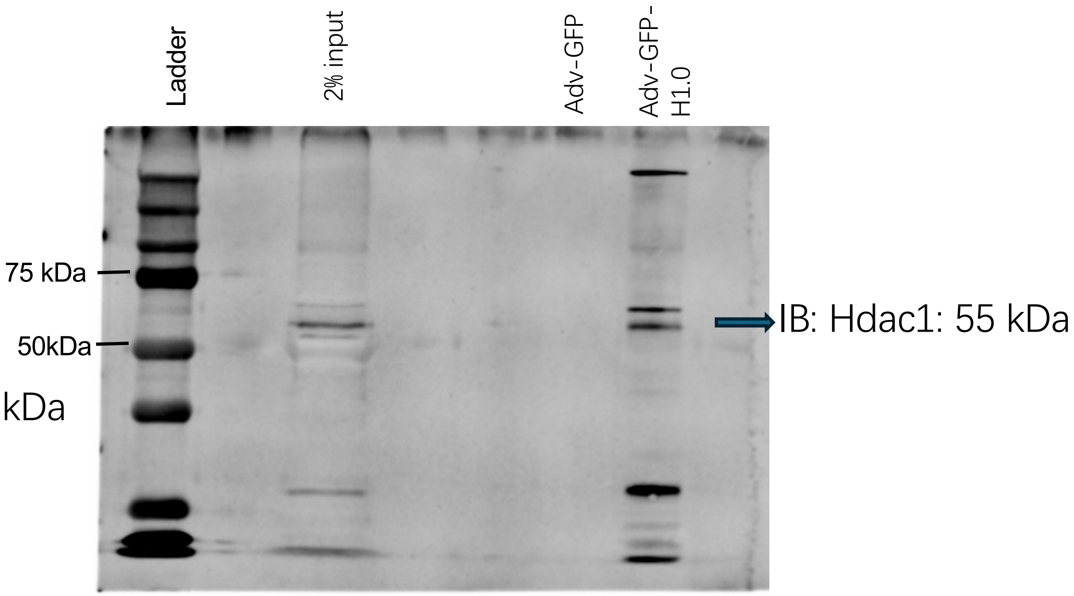

Source Data for: Hu et al. Histone H1.0 Couples Cellular Mechanical Behaviors to Chromatin Structure

Figure 4i

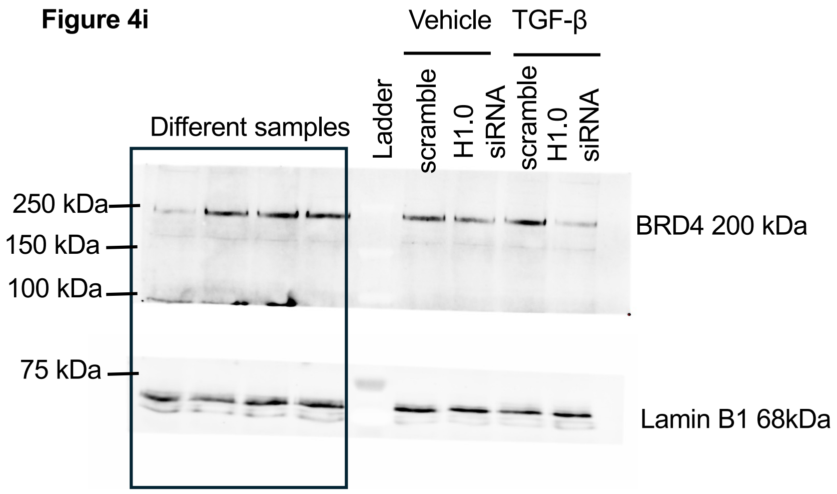

Source Data for: Hu et al. Histone H1.0 Couples Cellular Mechanical Behaviors to Chromatin Structure
