# Supplementary figures and images for: Histone H1.0 couples cellular mechanical behaviors to chromatin structure
Source: Nat Cardiovasc Res. 2024 Apr 10;3(4):441–59. doi: 10.1038/s44161-024-00460-w (PMC11101354; doi:10.1038/s44161-024-00460-w)

Figure 7b

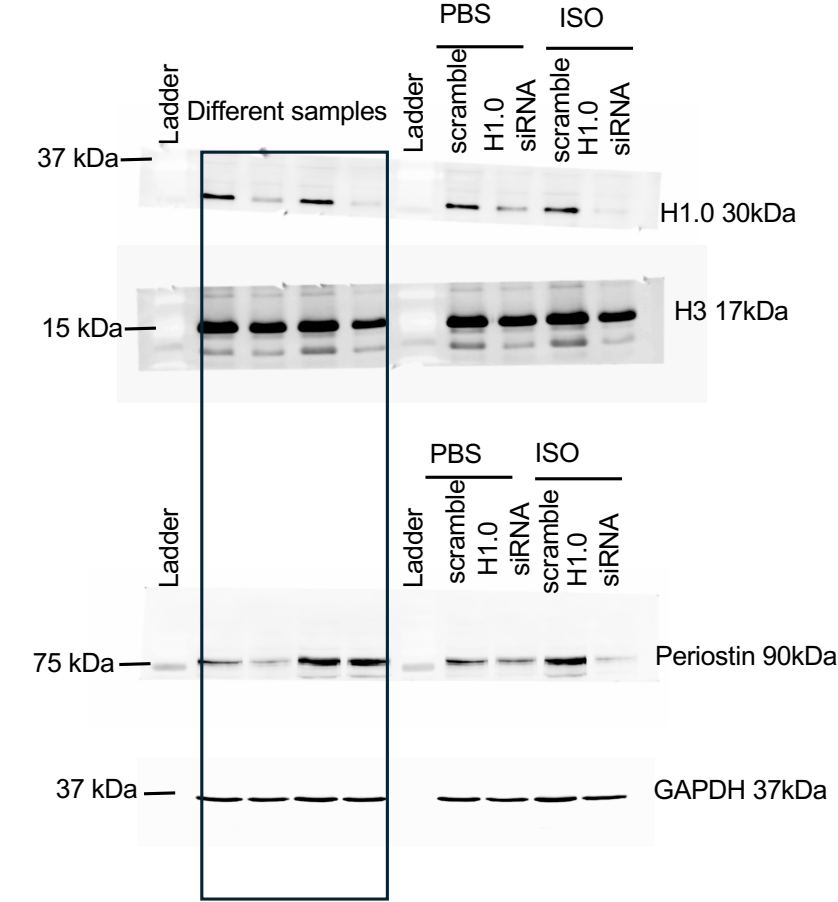

Figure 7f

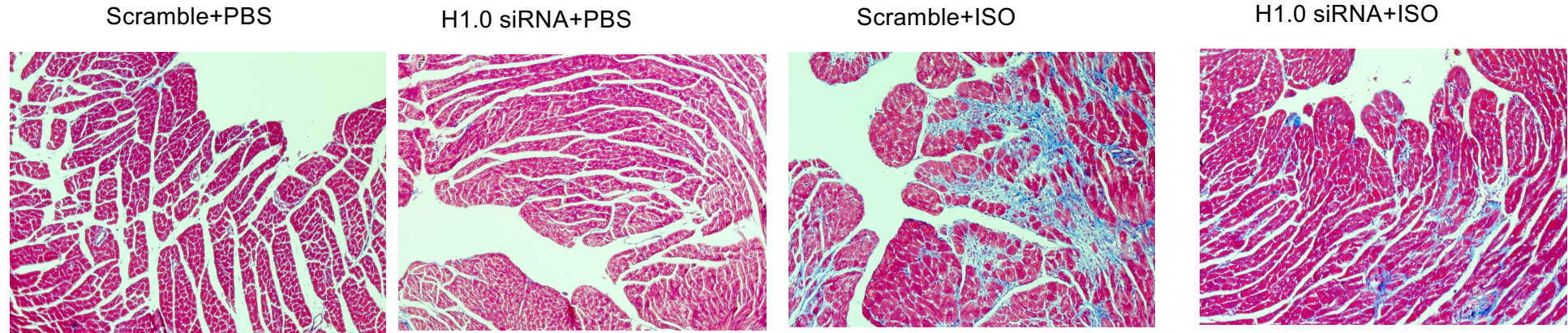

Supplement: Supplementary file 15 — Unprocessed images and blots in Fig. 7. [file 44161_2024_460_MOESM15_ESM.pdf]

Extended Data Figure 7a

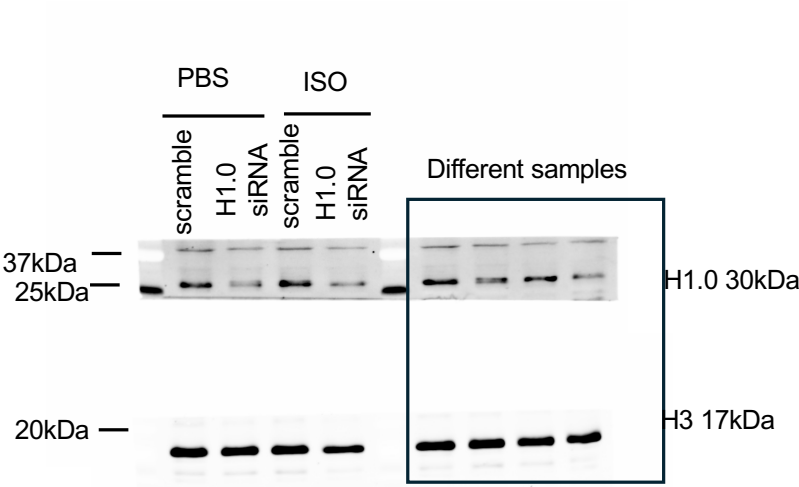

Extended Data Figure 7b

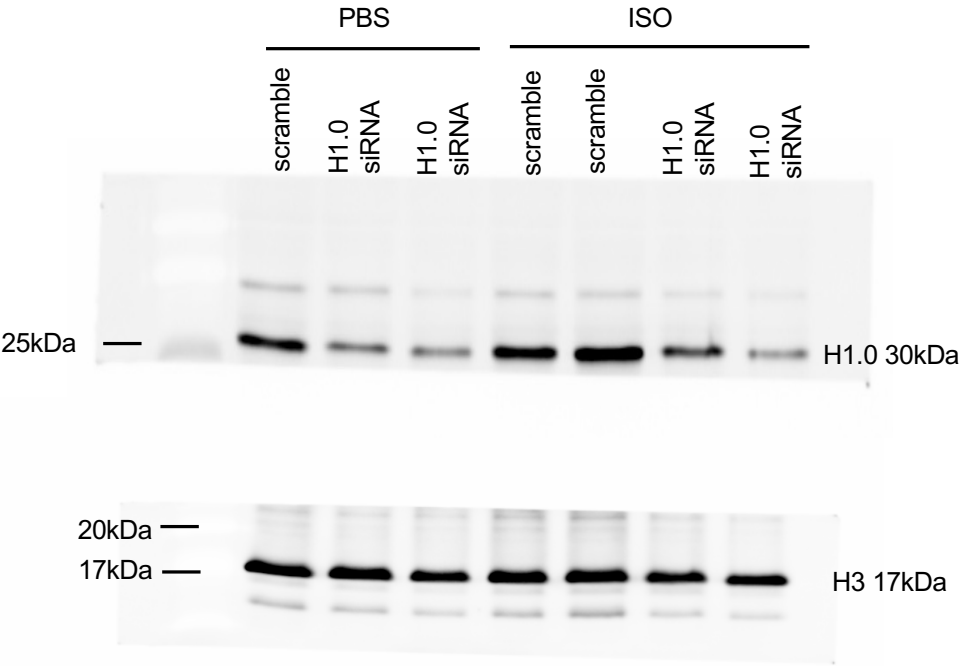

Supplement: Supplementary file 26 — Unprocessed images and blots in Extended Data Fig. 7. [file 44161_2024_460_MOESM26_ESM.pdf]
