# Supplementary material for: Histone H1.0 couples cellular mechanical behaviors to chromatin structure
Source: Nat Cardiovasc Res. 2024 Apr 10;3(4):441–59. doi: 10.1038/s44161-024-00460-w (PMC11101354; doi:10.1038/s44161-024-00460-w)
Supplement: Supplementary file 21 — Unprocessed images and blots in Extended Data Fig. 4. [file 44161_2024_460_MOESM21_ESM.pdf]

Extended Data Figure 4a

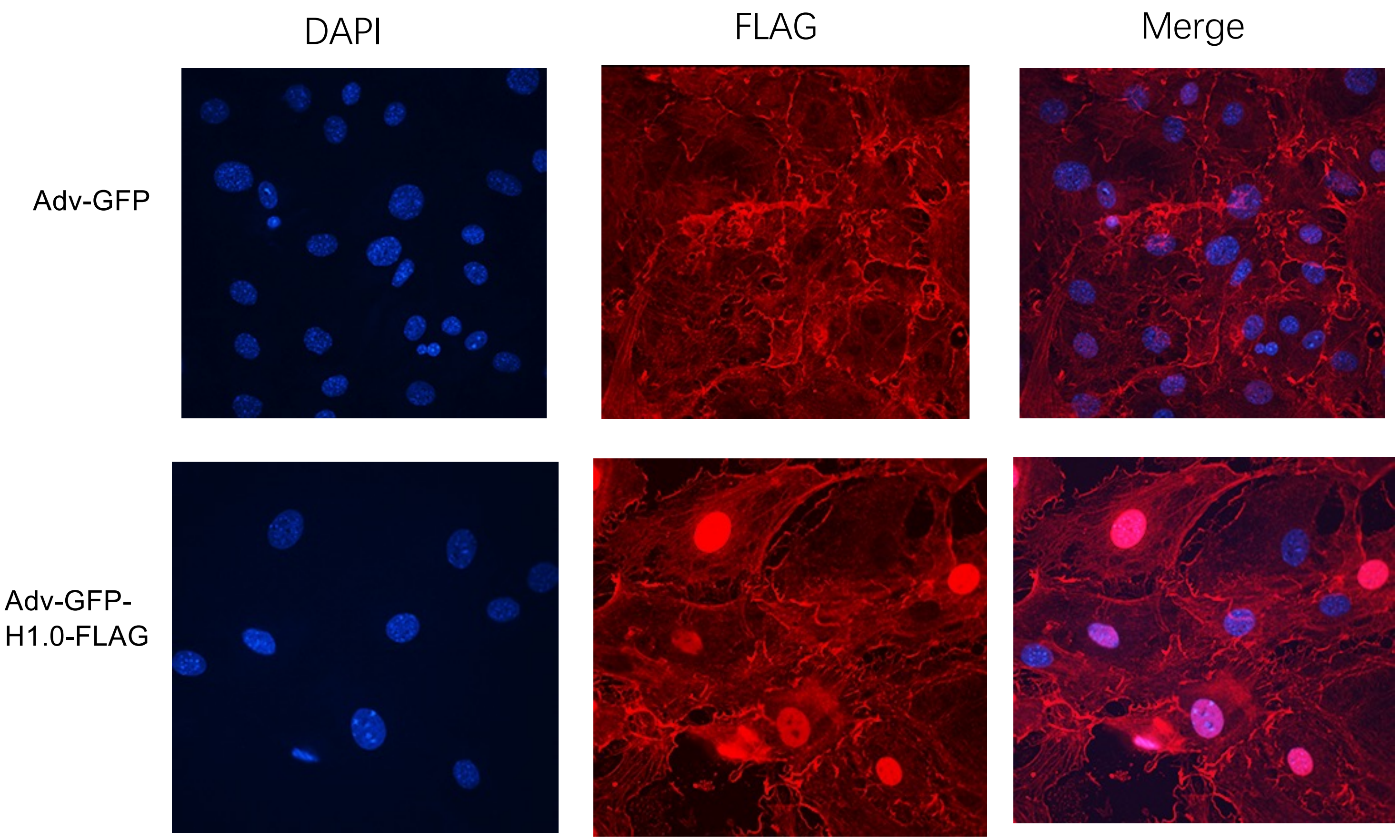

Source Data for: Hu et al. Histone H1.0 Couples Cellular Mechanical Behaviors to Chromatin Structure

Extended Data Figure 4b

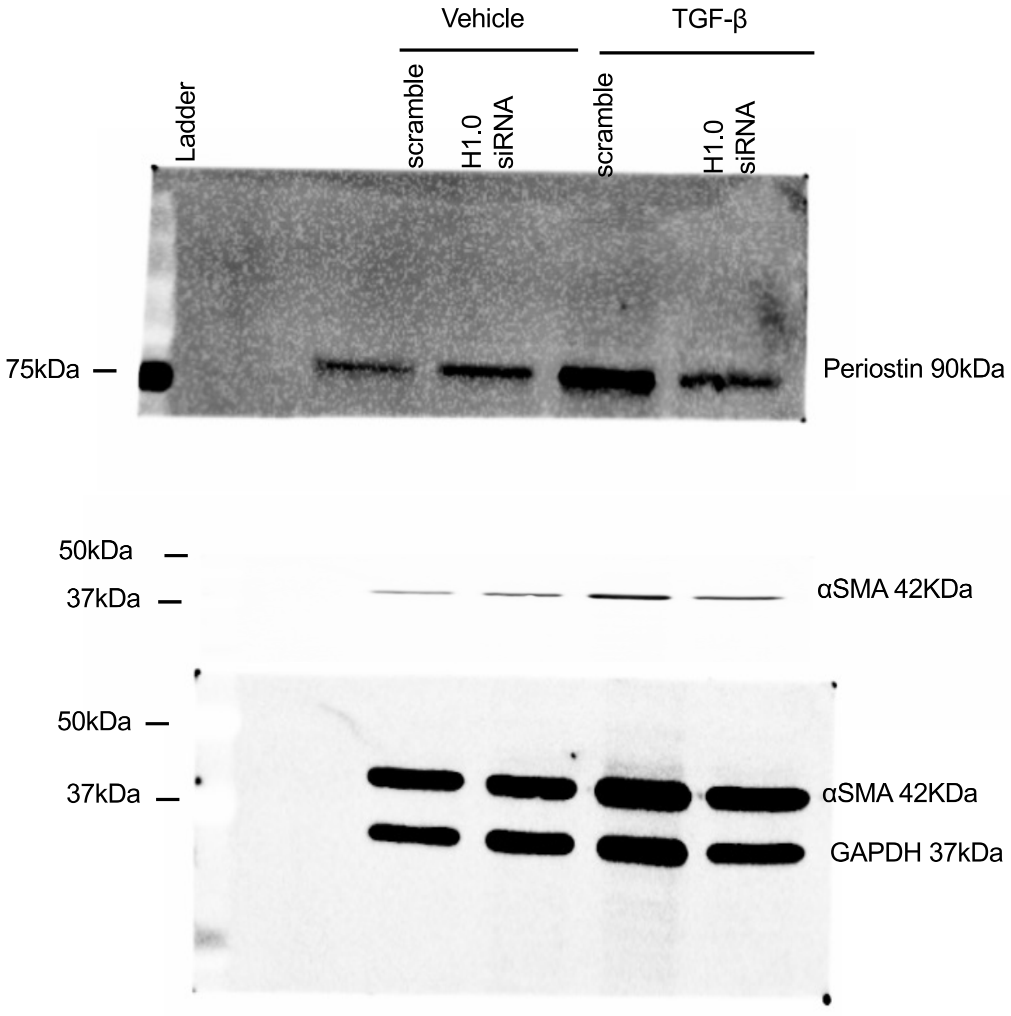

Upper band: αSMA-42kDa
Bottom band: GAPDH-37KDa
After the first day, the membrane was exposed to test αSMA, Subsequently, the same membrane was incubated overnight with the GAPDH primary antibody to measure GAPDH band the next day.

Source Data for: Hu et al. Histone H1.0 Couples Cellular Mechanical Behaviors to Chromatin Structure

Extended Data Figure 4c

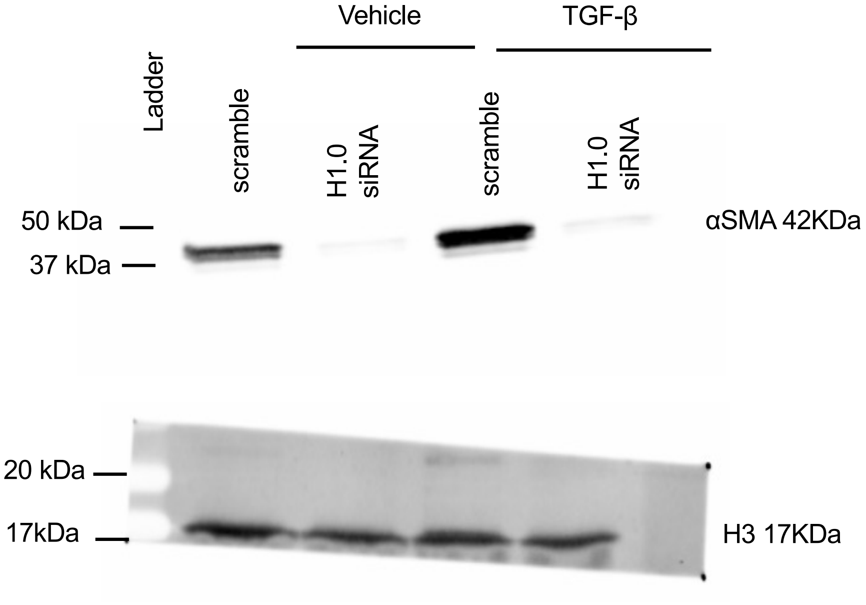

Extended Data Figure 4d

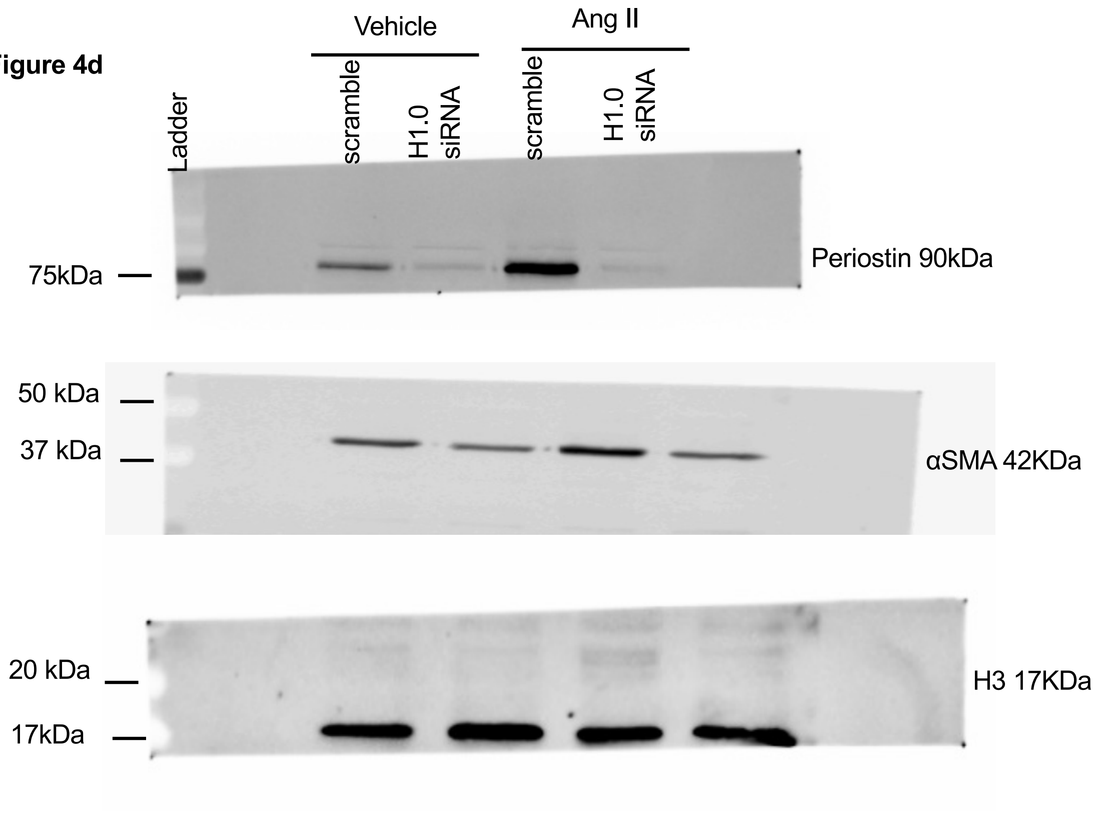

Source Data for: Hu et al. Histone H1.0 Couples Cellular Mechanical Behaviors to Chromatin Structure

Extended Data Figure 4f

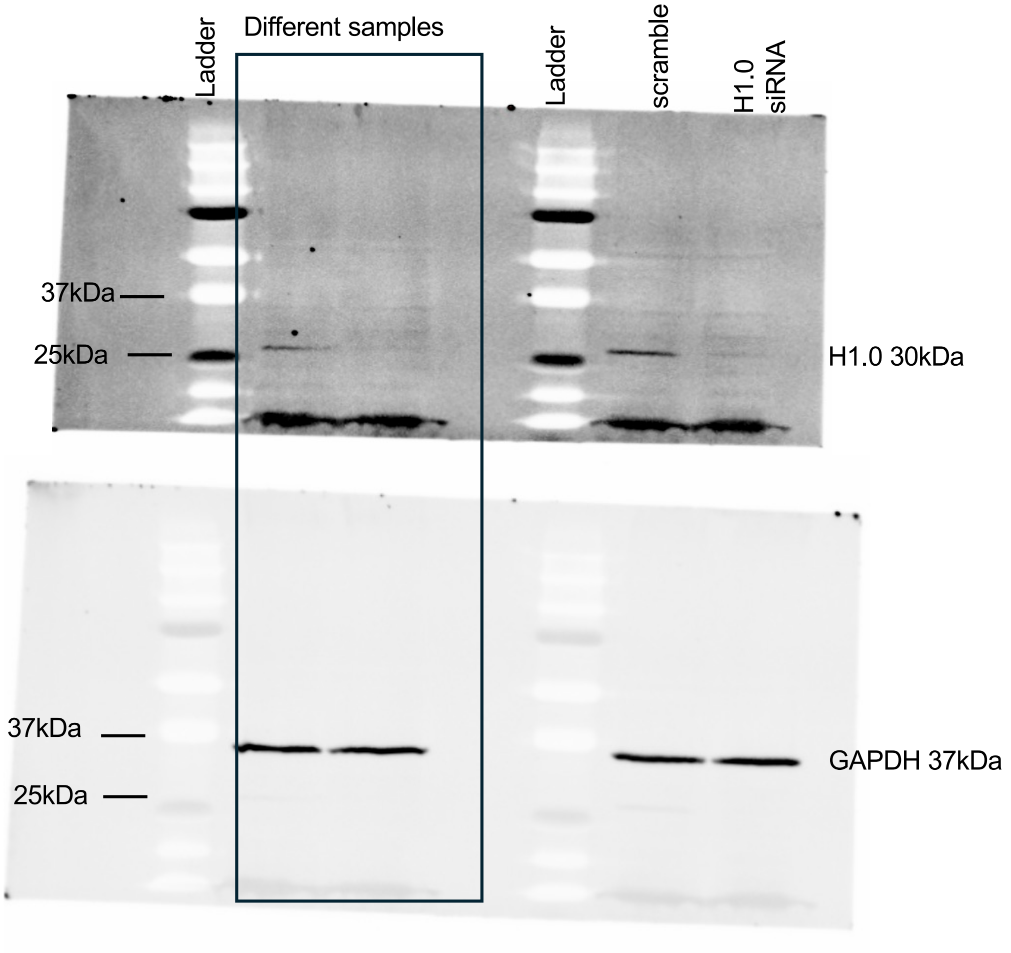

Source Data for: Hu et al. Histone H1.0 Couples Cellular Mechanical Behaviors to Chromatin Structure
