# Supplementary material for: Histone H1.0 couples cellular mechanical behaviors to chromatin structure
Source: Nat Cardiovasc Res. 2024 Apr 10;3(4):441–59. doi: 10.1038/s44161-024-00460-w (PMC11101354; doi:10.1038/s44161-024-00460-w)
Supplement: Supplementary file 23 — Unprocessed images and blots in Extended Data Fig. 5. [file 44161_2024_460_MOESM23_ESM.pdf]

Extended Data Figure 5c

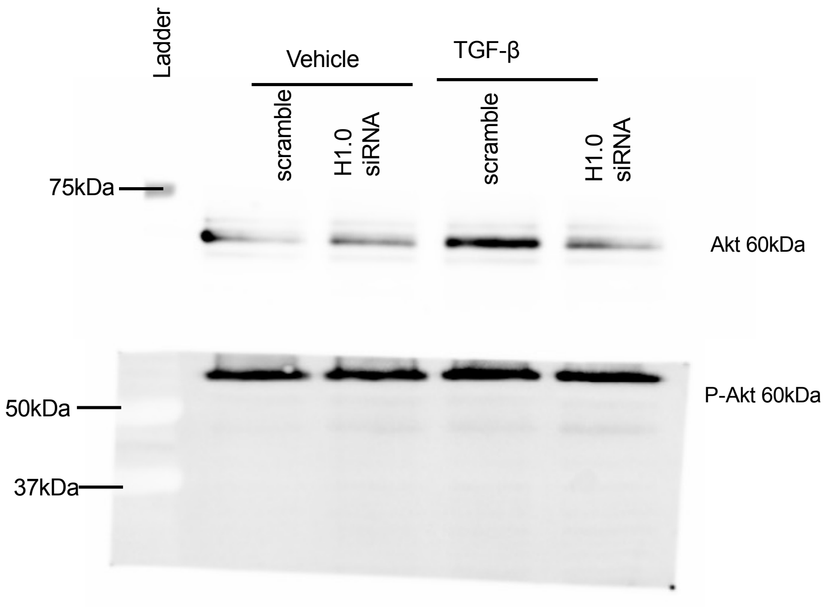

Extended Data Figure 5d

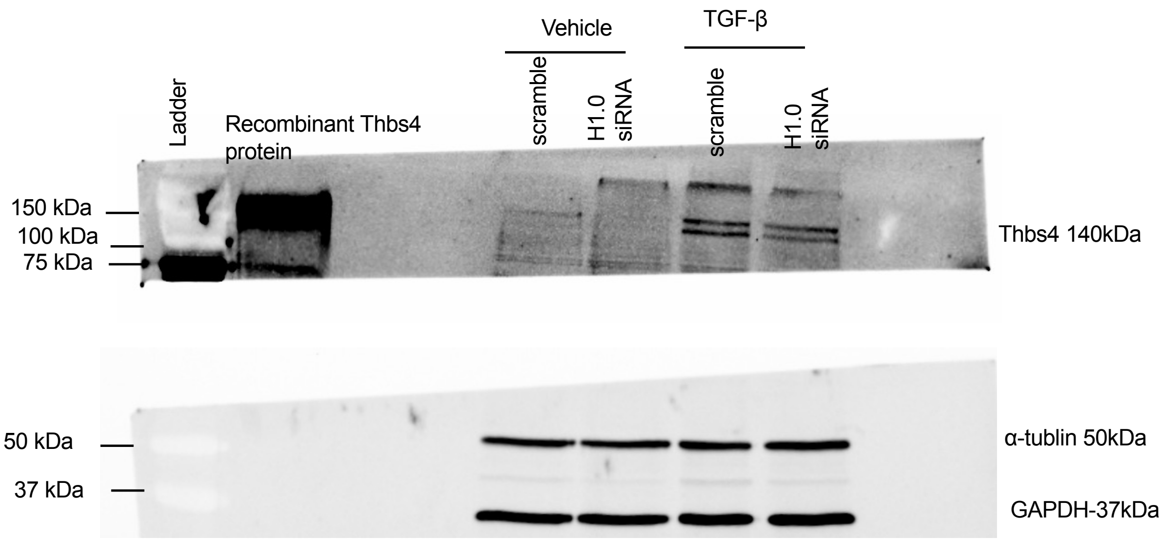

Source Data for: Hu et al. Histone H1.0 Couples Cellular Mechanical Behaviors to Chromatin Structure

Extended Data Figure 5e

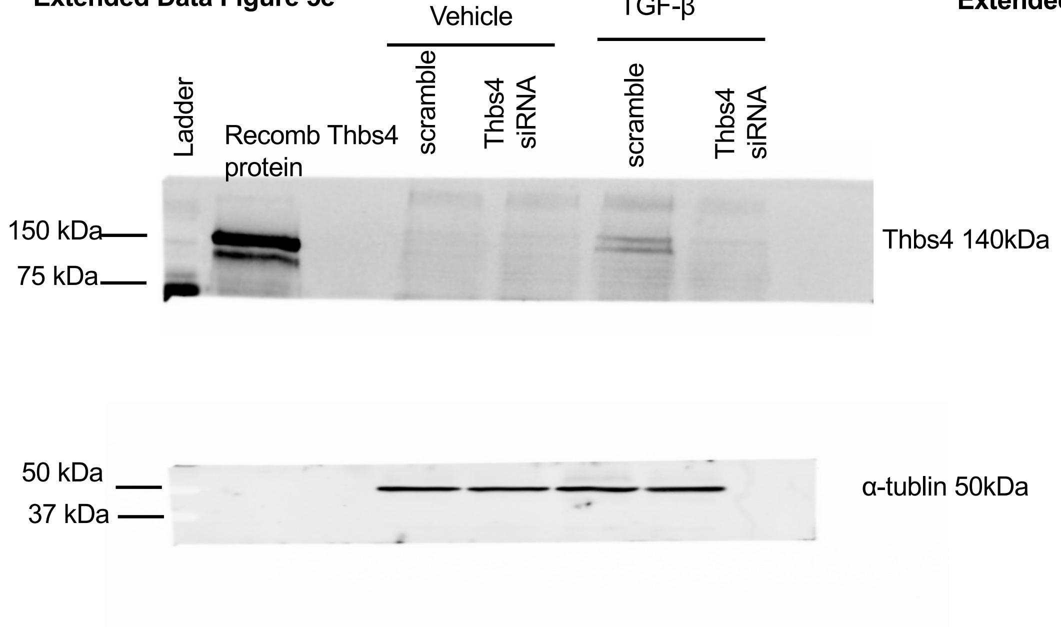

Extended Data Figure 5f

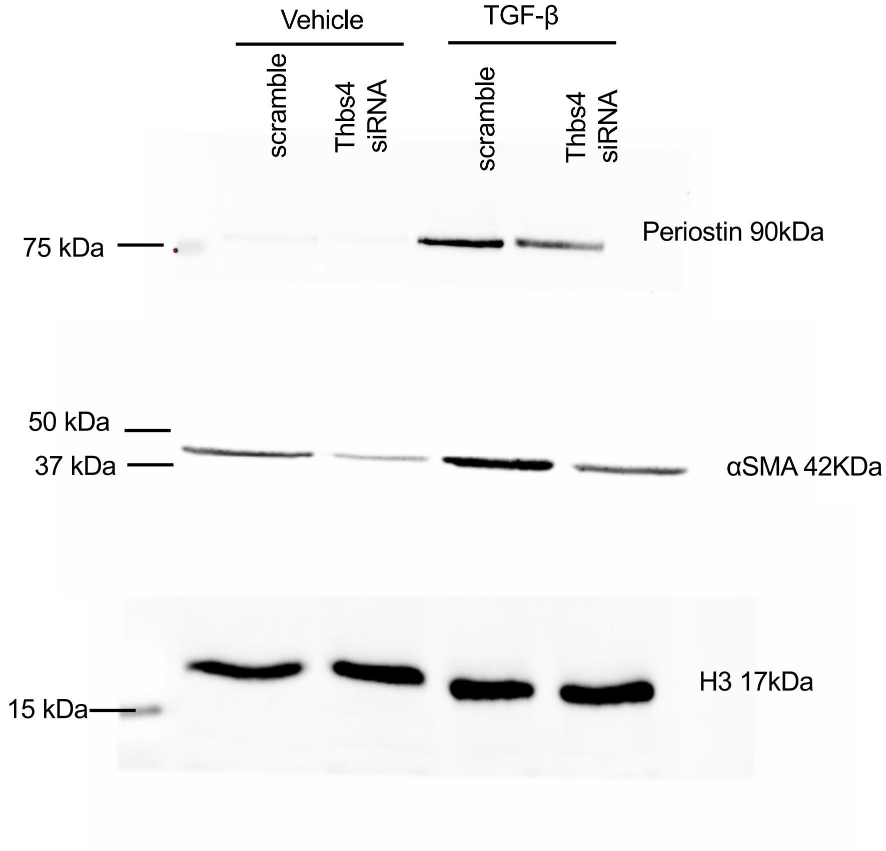

Source Data for: Hu et al. Histone H1.0 Couples Cellular Mechanical Behaviors to Chromatin Structure

Extended Data Figure 5h

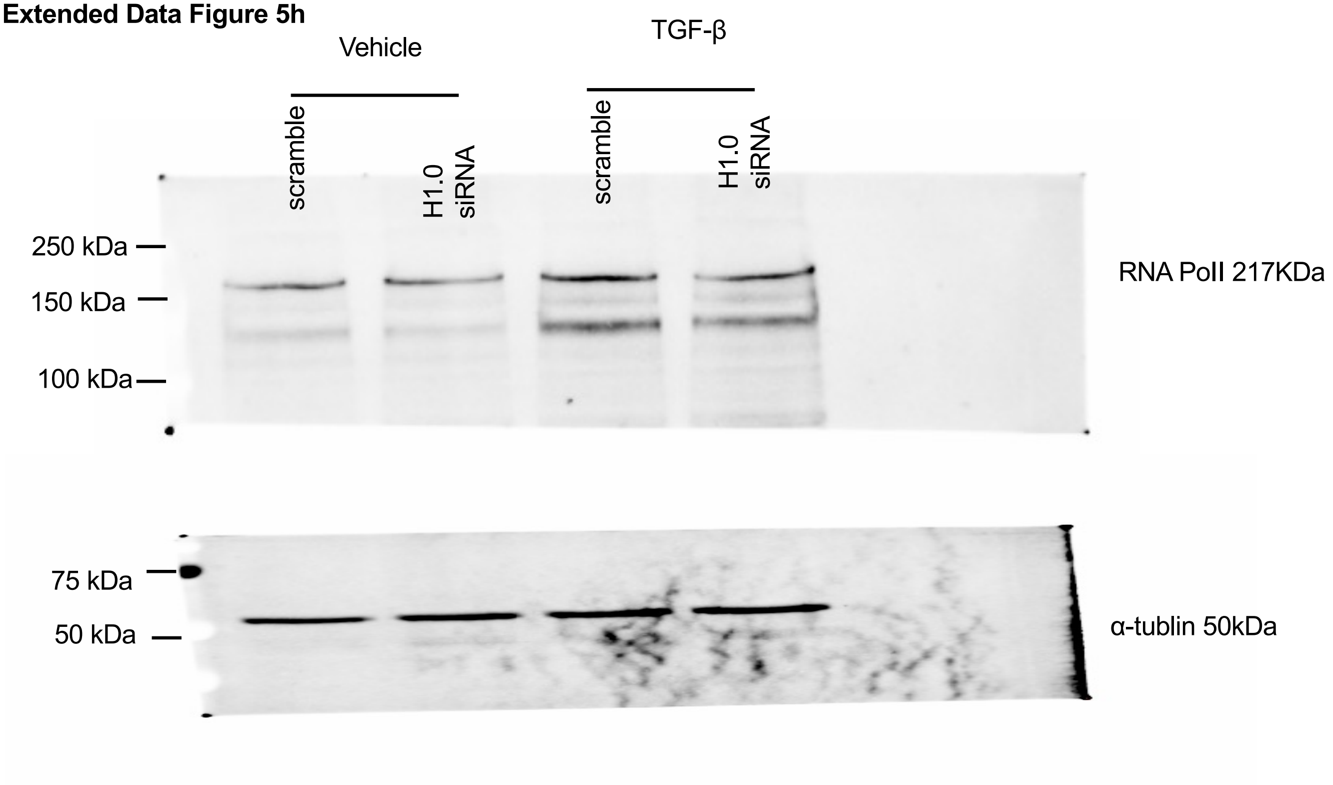

Source Data for: Hu et al. Histone H1.0 Couples Cellular Mechanical Behaviors to Chromatin Structure
